# Monitoring of antimicrobial resistance genes and influenza viruses in avian-populated water bodies

**DOI:** 10.1093/sumbio/qvaf013

**Published:** 2025-07-21

**Authors:** Kata Farkas, Margaret E Knight, Nick Woodhall, Rachel C Williams, Wannipa Seerung, Reshma Silvester, Rachel Abbey, Matthew J Wade, Lorenzo Cattarino, Tom White, Davey Jones

**Affiliations:** School of Environmental and Natural Sciences, Bangor University, Bangor, Gwynedd LL57 2UW, United Kingdom; School of Environmental and Natural Sciences, Bangor University, Bangor, Gwynedd LL57 2UW, United Kingdom; School of Environmental and Natural Sciences, Bangor University, Bangor, Gwynedd LL57 2UW, United Kingdom; School of Environmental and Natural Sciences, Bangor University, Bangor, Gwynedd LL57 2UW, United Kingdom; Public Health Group, Verily Life Sciences, Dallas, TX 75019, United States; School of Environmental and Natural Sciences, Bangor University, Bangor, Gwynedd LL57 2UW, United Kingdom; School of Environmental and Natural Sciences, Bangor University, Bangor, Gwynedd LL57 2UW, United Kingdom; Chief Data Officer Group, UK Health Security Agency, London E14 4PU, United Kingdom; Chief Data Officer Group, UK Health Security Agency, London E14 4PU, United Kingdom; Chief Data Officer Group, UK Health Security Agency, London E14 4PU, United Kingdom; Chief Data Officer Group, UK Health Security Agency, London E14 4PU, United Kingdom; School of Environmental and Natural Sciences, Bangor University, Bangor, Gwynedd LL57 2UW, United Kingdom

**Keywords:** antimicrobial resistance (AMR), environmental monitoring, faecal source tracking, influenza, public health, zoonosis

## Abstract

Monitoring zoonotic and multi-species pathogens is challenging due to the resources required to sample individual animals. In this study, we propose the use of DNA/RNA detection methods to assess the prevalence of bacterial, antimicrobial resistance (AMR), and viral markers in 19 water sites in England and Wales where close contact occurs with avian species. For bacteria and AMR monitoring, we utilized high-throughput quantitative polumerase chain reaction (HT-qPCR), which revealed a consistent abundance of AMR genes and mobile genetic elements, with aminoglycoside-resistance gene *aadA7* being most prevalent. Elevated levels of multidrug resistance genes were detected at two sites, potentially linked to wildlife rather than anthropogenic factors. The *intl3* gene was a dominant integron, supporting its use as a marker for AMR prevalence. We also developed a streamlined pipeline (including sampling, sample process and pathogen identification/quantification) for influenza prevalence assessment. The detection of influenza virus coincided with reported avian influenza outbreak in wild birds. The frequent detection of human (crAssphage) and bird-associated (*Catellicoccus marimammalium*) markers suggested that they provide a robust framework for assessing faecal pollution in water bodies. This study demonstrates how molecular monitoring can track emerging pathogens simultaneously and identify pollution sources, providing a valuable approach for the surveillance of zoonotic threats.

**Sustainability Statement**

This study directly supports the United Nations Sustainable Development Goal of One Health by providing an innovative environmental monitoring framework for tracking antimicrobial resistance (AMR) genes and influenza viruses in avian-associated water bodies. One Health emphasizes the interconnectedness of human, animal, and environmental health, and our research aligns with this goal by addressing the critical issue of zoonotic disease surveillance and environmental contamination.

Using high-throughput quantitative PCR and other molecular detection techniques, we present a sustainable method for early detection of public health threats that reduces the need for intensive animal sampling and enhances surveillance efficiency. Our findings highlight avian populations’ role in spreading AMR genes and influenza viruses, reinforcing the importance of integrated monitoring strategies. The identification of pollution sources through faecal markers provides insights for targeted mitigation strategies, thereby supporting ecosystem health and water quality management.

Our research fosters a proactive approach to disease prevention, aligning with global efforts to combat emerging diseases threatening human and animal health. Our work underscores the importance of interdisciplinary collaboration in achieving One Health objectives and promoting resilient public health infrastructure.

## Introduction

Migratory birds can spread microorganisms and viruses globally across all continents (Altizer et al. 2011). Some of those microorganisms can be harmful for birds and mammals, including humans (Verhagen et al. 2021). Hence, the introduction of a new pathogen from birds can result in large epidemics or pandemics, which threaten wildlife, agriculture, and human health (Peacock et al. 2025). The annual migratory movements of birds facilitate the even distribution of certain pathogens, resulting in minimal variation over time (Jourdain et al. 2007, Chen et al. 2024). However, due to the rapid evolution of microorganisms and viruses, novel pathogenic strains which may be more harmful or able to infect different species, often emerge (Vandegrift et al. 2010, Gamarra-Toledo et al. 2023). Migratory birds host numerous pathogens that have been known to be zoonotic, including arthropod-borne pathogens (arboviruses, West Nile virus, *Borrelia* causing Lyme disease) and various respiratory and enteric diseases caused by viral (influenza A virus) and bacterial (*Salmonella* spp. *Campylobacter jejuni, Enterococcus* spp., *Mycobacterium* spp*., Chlamydia psittaci*) pathogens (Reed et al. 2003, Weaver and Reisen 2010).

Recent studies have shown that the pathogens birds harbour can be antimicrobial resistant (AMR) and hence migratory birds contribute to the global spread of AMR pathogens, potentially introducing such pathogens to previously unaffected regions (Sandegren et al. 2018, Franklin et al. 2020). Due to urbanization, birds are living in close proximity to humans and exposed to bacteria present in agricultural runoff, wastewater, waste, and debris. These pathogens may not infect the hosting birds but yet survive in the gut of birds and be transmitted through their movements (Höfle et al. 2020, Martín-Vélez et al. 2024). Climate change can disrupt the migratory routes of birds leading to some species to travel to new areas and that can further contribute to the spread of AMR and other diseases. This highlights the interconnectedness of ecosystems and the need for comprehensive and proactive monitoring that considers the role of birds and potentially other wild animals in the spread of AMR and other emerging pathogens.

Among the health risks associated with bird-borne zoonosis, avian influenza viruses (AIV) have been the greatest concern (Li et al. 2019). These enveloped viruses have a segmented RNA genome, and co-infections of different subtypes often result in reassortment and the emergence of novel variants potentially harmful to mammals (Mostafa et al. 2018, Lin et al. 2023). Most reassortment events, which can lead to a shift in host species, involve hemagglutinin (H) and neuraminidase (N) genes. These genes encode the two primary surface proteins of the influenza virus and play key roles in host specificity and immune response. The variations in these genes form the basis of influenza A virus subtyping. For example, the 1918 Spanish flu and the 2009 swine flu pandemics were both instigated by novel subtypes of H1N1 reassorted from human, swine, and avian origins (Smith et al. 2009, Anhlan et al. 2011).

The AIV subtypes encoding the H5 and H7 genes are substantially different in their pathogenicity (Liang 2023). The low pathogenicity AIV (LPAIV) H7 subtypes usually result in mild illness or remain asymptomatic. In contrast, the high pathogenicity AIV (HPAIV) H5 subtypes often cause mass mortality and morbidity in poultry and wild bird populations, leading to significant economic losses and disruptions in avian populations (Kalthoff et al. 2010). The H5N1 subtype has been under scrutiny due to the potential risk it presents to human health globally since 1997, when the first human case was identified (Mittal and Medhi 2007). Between 1997 and 2023, the H5N1 subtype has been responsible for ∼1000 human cases and 463 deaths, with peaks in cases in 2006 and 2015 reported in 24 countries (CDC 2024a; WHO 2024). Most human cases were associated with poultry and originated in China, where the seropositive for H5N1 was determined as 2.45% (Qi et al. 2020). Between 2022 and 2024, the number of outbreaks linked to avian influenza virus strain H5N1 increased significantly among birds, mainly waterfowl and poultry, and mammalian species, e.g. seals, otters, dolphins, foxes, racoons, mice, and cats, globally (Leguia et al. 2023, Puryear et al. 2023). The virus has more recently spilled over into dairy cows in North America, resulting in 70 human cases, including one death among mostly farm workers in 13 states in the US and one case in Canada between March 2024 and March 2025 (Dyer 2024, CDC 2024b). Effective monitoring of the virus is essential to maintain low infectivity rates in both humans and animals.

To effectively prevent and mitigate zoonotic outbreaks, avian-associated pathogens with pandemic potential, such as avian flu and AMR bacteria, should be continuously monitored. Current monitoring of bird-associated diseases is largely limited to testing dead and sick animals during outbreaks, which is both resource-intensive and restricted to easily accessible populations (Yang et al. 2023). The surveillance of wild water bird populations would be particularly important to protect poultry and human health; however, such monitoring is not employed in most countries. Current testing regimen targets notifiable diseases that are already in circulation, and the novel and potentially emerging pathogens are often overlooked. A more inclusive approach would be necessary for the assessment of pathogen circulation to mitigate the spread of diseases.

Many pathogens of public health interest and emerging concern can colonize the avian gastrointestinal tract and are therefore shed into the water during feeding or via faeces (Smith et al. 2020). Hence, monitoring of environmental matrices (e.g. water sediment) may have potential to track pathogens infecting waterbirds (Ahrens et al. 2023). Traditionally, most research studies have relied on culturing-based methods to isolate avian influenza and AMR-carrying pathogens (Deboosere et al. 2011, Franklin et al. 2020, Höfle et al. 2020, Hubbard et al. 2023). While these methods are sensitive and precise, they are very time consuming, especially for viruses, where culturing may take weeks to perform and requires specialized equipment. Culturing also excludes pathogens that cannot be grown *in vitro* and those that are in a viable but not culturable state and hence underestimates health risks (Liu et al. 2023). Furthermore, pathogen cultures pose a health risk, requiring certain work to be conducted in a biosafety level 3/containment level 3 (BSL3/CL3) environment. To overcome these challenges, environmental nucleic acid (eNA) monitoring can be applied to detect genetic material of pathogens directly from soil, water, sediment, or air, eliminating organism isolation for building safe and efficient surveillance systems. To date, the eNA approach has proven effective for the tracking a wide range of species (e.g. fish, insects, and bacteria) through their genetic material in the environment supporting wildlife conservation and management decisions (Rees et al. 2014, Ruppert et al. 2019). However, these approaches remain underutilized for monitoring bird-associated zoonotic pathogens, especially emerging viruses and bacteria.

**Figure 1.**
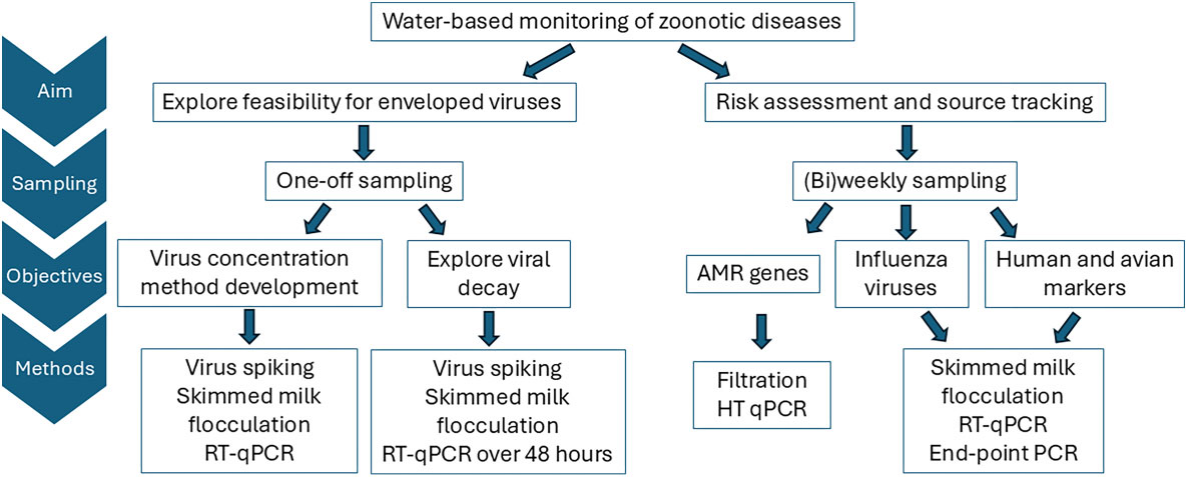
Flowchart of the assay design.

The aim of this study was to explore the usefulness of eNA monitoring for viral and bacterial markers in surface water bodies frequented by migratory birds across England and Wales to support the risk assessment and management of zoonotic and AMR pathogens. We focused influenza virus as an emerging zoonotic health threat, a panel of AMR markers and human and bird-associated faecal markers to explore the potential of source tracking. We utilized quantitative and endpoint PCR to assess pathogen distribution and prevalence. We also established a standardized sampling protocol and analytical workflow for monitoring AMR genes and influenza viruses (including method validation) in surface water bodies for animal and public health surveillance (Fig. 1).

## Materials and methods

### Sampling sites

**Figure 2.**
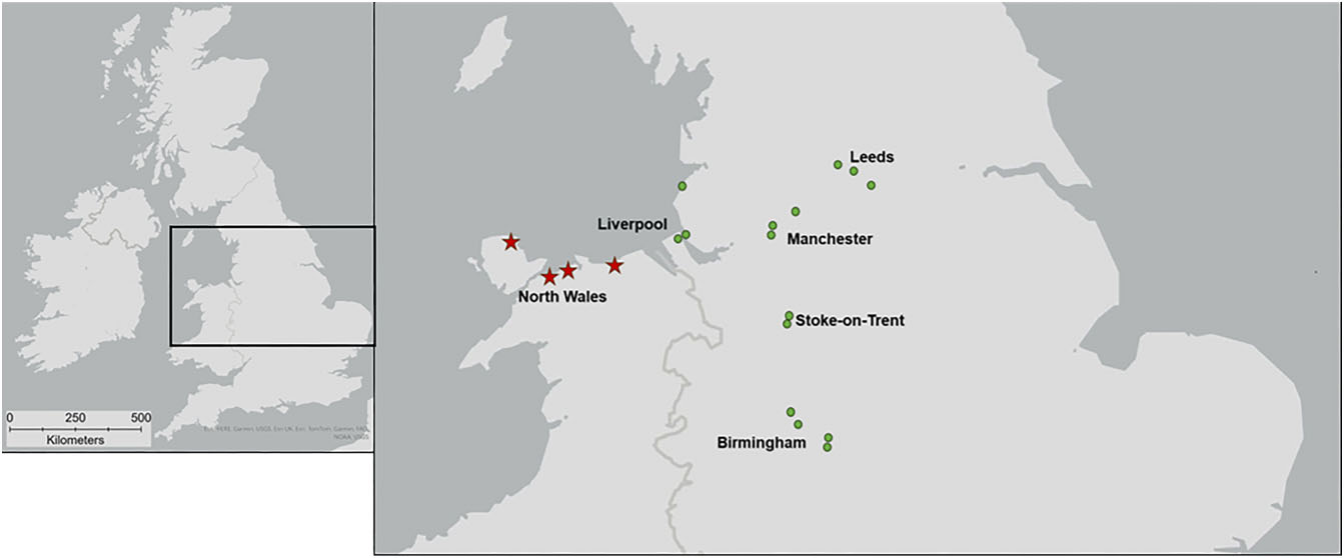
Map showing the sampling sites in England (green circles) and in Wales (red stars).

Sampling sites were selected in collaboration with the British Trust of Ornithology, comprising 15 sites in England and 4 sites in North Wales (Fig. 2; Table S1). We selected urban sites based on the following criteria using spatial analysis: (i) classified as still water, (ii) within 50 m of a road for accessibility, (iii) within 1 km of a recorded wild bird avian influenza infection (data provided by APHA and BTO), and (iv) within 150 miles driving distance from Bangor University to avoid nucleic acid decay during prolonged transport.

### Sampling approach

The main sampling campaign was conducted weekly between 15th January and 21st March 2024, across all 19 sites (Table S1, *n* = 188). Additional longitudinal sampling was conducted fortnightly at four North Wales sites: Brickfields Pond (9th November 2023–4th January 2024; *n* = 4), Llanfairfechan (29th August 2023–4th January 2024; *n* = 9), and both Parc Menai and the Spinnies (3rd March 2023–4th January 2024; *n* = 16). At each sampling event, 11 l water samples were collected in polypropylene containers at the edge of the water body. Supplementary samples (*n* = 4) were collected from the North Wales sites for method validation and viral recovery and decay studies as detailed below. All samples were transported chilled to Bangor University laboratories and process started on the same day.

### Water quality testing

Water samples taken during the 10-week monitoring (*n* = 233) were tested for pH, electrical conductivity, turbidity, ammonium, and orthophosphate levels as described elsewhere (Hillary et al. 2021) to evaluate any major temporal changes in water quality over the study period.

### High-throughput quantitative PCR-based detection of ARGs, MGEs, and taxonomic markers

A 200–1000 ml aliquot of 204 water samples (Table S2) were filtered through 0.22 µm polyethersulfone (PES) membranes (Gilson, Middleton, WI, USA) and the membranes stored at −80°C, and sent to Resistomap Oy (Helsinki, Finland). The DNA extraction, quality control and high-throughput quantitative PCR (HT-qPCR) were performed by Resistomap Oy. Filter concentrates from 204 water samples were subject to DNA extraction using the DNeasy PowerWater Kit (Qiagen, Hilden, Germany) and the resulting DNA was quantified using NanoDrop One (Thermo Fisher Scientific, Waltham, MA, USA). The DNA concentration was adjusted to 10 ng/µl prior to analysis. Extracts were then analysed for targeted genes using high throughput qPCR at using established primers (Muziasari et al. 2017). The qPCR assay had 92 targets, including 74 AMR genes [including six multidrug-resistance (MDR) related gene fragments], 10 mobile genetic elements (MGEs), 5 taxonomic markers (pathogens), 2 metal-resistance genes, and the 16S rRNA gene to estimate total bacterial load (Table S3). The six MDR genes were selected as described earlier (Stedtfeld et al. 2018). For the qPCR run, already published conditions were used (Muziasari et al. 2017) on a SmartChip™ Real-Time PCR system (TakaraBio USA, San Hose, CA, USA). Briefly, the SmartChip has 5184 reaction wells with a volume of 100 nl and filled using the SmartChip Multisample Nanodispenser (TakaraBio, USA). Each reaction consisted of SmartChip TB Green Gene Expression Master Mix as recommended by the manufacturer, 20 nl of template DNA, 300 nM of forward and reverse primers and qPCR water. The PCR consisted of 10 min denaturation step at 95°C followed by 40 cycles of 30 s denaturation at 95°C and 30 s annealing at 60°C and melting curve analysis. Each DNA sample was analysed in triplicate with at least two replicates to produce a cycle threshold (CT) of 27 or lower for the sample to be considered positive. The relative abundances of the detected genes in comparison to the 16S rRNA gene were determined using the ΔCT method, based on the average CT values obtained from three technical replicates. The relative abundances were expressed as gene copies (gc) per 16S rRNA gc.

### Virus concentration method validation by spiking

To assess the recovery of influenza virus during sample concentration, quadruplet water samples taken at Llanfairfechan, the Spinnies, Brickfields Pond, and at Parc Menai, North Wales were spiked with H3N2 influenza A virus (VR-1881, ATCC, Manassas, VA, USA), which was diluted to reach a final concentration of ∼10^6^ gc/l in the spiked samples, enabling quantification in samples with 0.01% recovery. We chose this strain to represent avian flu as it is a readily culturable BSL2 pathogen, which is available commercially at high titres. Samples were then concentrated using the skimmed milk flocculation (SMF) method, as described below. To ensure the spiked virus is thoroughly mixed, the sample volume was reduced to 3 l. The SMF method is suitable for the concentration of samples with 0.5–10 l volumes, hence, we anticipated that the reduction in sample volume would have a negligible effect (Calgua et al. 2013, Donchev et al. 2024). The concentrates were extracted and tested using RT-qPCR as described below.

### Viral decay assessment by spiking

To determine the stability of the influenza virus in surface water, 3 l water were samples collected from North Wales at Brickfields Pond, Park Menai, Llanfairfechan, and the Spinnies and were spiked with H3N2 influenza A virus in six replicates as detailed above. Two replicates were processed with the SMF method described below immediately after spiking and two duplicates were processed after 24 and 48 h incubation at room temperature. The concentrates were extracted and tested using RT-qPCR as described below.

### Sample concentration

All collected water samples were concentrated using the SMF as described previously (Calgua et al. 2013, Farkas et al. 2024). Sample volume was 10 l for the weekly monitoring samples and 3 l for the spiking experiments. In brief, sample pH and conductivity were adjusted to 3.5 and 1.5 mS/cm, respectively, followed by the addition of skimmed milk to reach the final concentration of 0.01%. Samples were then stirred for 8 h and sedimented for 8 h followed by the removal of supernatant. The pellet was resuspended in phosphate saline buffer pH 7.4 to reach the final volume of 2–14 ml (depending on sample turbidity).

### Viral RNA extraction and concentration

Viral RNA was extracted from the 0.2 ml aliquots of the concentrates using the NucliSENS extraction kit (BioMerieux, Marcy-l'Étoile, France) on a KingFisher 96 Flex system (Thermo Fisher Scientific) as described previously (Kevill et al. 2022). In brief, the samples were mixed with 0.8 ml NucliSENS easyMAG Lysis Buffer for 10 min followed by binding to NucliSENS easyMAG Magnetic Silica beads for another 10 min. Then, the beads binding to DNA/RNA were washed with 0.4–0.5 ml NucliSENS easyMAG Extraction Buffers 1–3, as per manufacturer’s protocol. The purified RNA/DNA were eluted in 0.1 ml NucliSENS easyMAG Extraction Buffer 3 at 60°C for 5 min. For each concentrated sample, eight replicates were extracted. For enrichment before subtyping, the GeneJET RNA Cleanup and Concentration Micro Kit (Thermo Fisher Scientific) was used, where 8 × 50 µl of the RNA extract was concentrated to 20 µl, following the manufacturer’s protocol.

### In house DNA/RNA quantification and influenza subtyping

We used RT-qPCR for the typing and quantification of influenza A viruses targeting the segment 7 matrix protein 2 gene (InfA assay) on all samples (*n* = 233) as described previously (Shu et al. 2021, Farkas et al. 2022). In brief, the 20 µl reaction mix contained TaqMan Viral 1-step RT-qPCR master mix (Applied Biosystems Inc., Foster City, CA, USA) with 1 µg bovine serum albumin (BSA), 16 nmol MgSO_4_, (Alfa Aesar, Heysham, UK) 0.5 µM forward (CAAGACCAATCYTGTCACCTCTGAC and CAAGACCAATYCTGTCACCTYTGAC), 1 µM reverse primers (GCATTYTGGACAAAVCGTCTACG and GCATTTTGGATAAAGCGTCTACG), 0.25 µM probe (FAM-TGCAGTCCT/ZEN/CGCTCACTGGGCACG-IAMkFQ) (IDT, Coralville, IA, USA), and 4 µl sample. The RT-qPCR run consisted of reverse transcription (50°C for 30 min) followed by deactivation-denaturation (95°C, 20 s) and 45 cycles of denaturation (95°C, 3 s) and annealing (60°C, 30 s). All extracts were run in duplicates with a dilution series of synthetic RNA as a standard and molecular-grade water as non-template control.

The positive samples were subtyped using endpoint PCR with the WHO PCR primers for the detection of H5N1 influenza viruses (WHO 2017) using the AmpliRUN^®^ Influenza A H5 RNA control (Vircell Microbiologists, Granada, Spain) as a positive control. We used the SuperScript™ III One-Step RT-PCR System with Platinum™ Taq High Fidelity DNA Polymerase (Invitrogen, Waltham, MA, USA) as a reaction mix, following the manufacturer’s instructions. The reaction mixes contained 0.5 µM of the H5-918F/H5-1166R primer pair or 1.5 µM of the H5-248-270F/H5-671-647R primers (WHO 2017). The RT-PCR consisted of reverse transcription (50°C for 30 min) followed by deactivation-denaturation (95°C, 2 min) and 40 cycles of denaturation (95°C, 15 s), annealing (55/52°C, 30 s) and elongation (68°C, 30 s), followed by final elongation (68°C, 5 min).

We also explored the prevalence of human and bird faecal pathogens targeting human gut-associated bacteriophage, crAssphage (*n* = 151) and gull-specific bacterium, *Catellicoccus marimammalium* (*n* = 146). Singleplex qPCR assays were used with the CPQ_056 and the gull4 primer and probe sets for crAssphage and *C. marimammalium*, respectively (Ryu et al. 2012, Stachler et al. 2017) as described previously (Farkas et al. 2022). In brief, the 20 µl reaction mix contained QuantiNova Probe PCR mix (Qiagen) with 1 µg BSA, 0.5 µM forward and reverse primers, 0.25 µM probe and 2 µl sample. The qPCR run consisted of denaturation (95°C, 2 min) and 40 cycles of denaturation (95°C, 15 s) and annealing (60°C, 1 min). All extracts were run in duplicates with a dilution series of synthetic RNA as a standard and molecular-grade water as non-template control.

### Data analysis

The virus and *C. marimammalium* concentrations derived from (RT-)qPCR were expressed as gc/µl nucleic acid extract and transformed to gc/l water (c) as


\begin{eqnarray*}
c = \textit{concentration}\ in\ \textit{nucleic}\ \textit{acid}\ \textit{eluent}*\frac{{\textit{eluent}\ \textit{volume}}}{{\textit{concentrate}\ \textit{volume}\ \textit{extracted}}}\\
{*}\frac{{\textit{total}\ \textit{volume}\ of\ the\ \textit{concentrate}}}{{\textit{sample}\ \textit{volume}}}.
\end{eqnarray*}

R version 4.2.1 (R Core Team, Austria) was used for data visualization. Bray–Curtis dissimilarity matrices, based on Hellinger-transformed ARG relative abundances, were applied for Principal Coordinates Analysis (PCoA; R4.2.1). PERMANOVA (i.e. Permutational Multivariate Analysis of Variance) and one-way ANOVA (i.e. Analysis of Variance) was used for comparisons between multiple groups, followed by pairwise analysis using Tukey’s test (R4.2.1). The water pH, conductivity, turbidity, ammonium, and orthophosphate concentrations along with the crAssphage and *C. marimammalium* concentrations were tested for normality using the Shapiro–Wilk test, which suggested that the data was not normally distributed (*P* < 0.001; SPSS V27, IBM, USA). Spearman’s correlation analysis was performed, for which we defined ‘strong significant correlations’ as ρ > 0.7, *P* < 0.01 (R4.2.1). A *t*-test was used to reveal significant differences in viral recovery rates in samples spiked with influenza virus (SPSS V27).

## Results

Samples were tested for basic physico-chemical properties as summarized in Table 1. No major trends or discrepancies over time were noted, suggesting that water quality was consistent over the study period.

**Table 1.** Summary of physico-chemical properties (mean ± standard error) of the water samples taken during the 10-week monitoring trial.

Site name	Region	pH	EC (mS/cm)	Turbidity (NTU)	Ammonium (mg/l)	Orthophosphate (mg/l)
Brickfields Pond	North Wales	8.17 ± 0.03	0.49 ± 0.00	2.7 ± 0.2	0.21 ± 0.14	0.02 ± 0.01
Llanfairfechan	North Wales	7.77 ± 0.06	4.47 ± 0.39	6.5 ± 0.6	0.15 ± 0.11	0.03 ± 0.01
Parc Menai	North Wales	7.99 ± 0.04	0.85 ± 0.04	5.0 ± 0.6	0.20 ± 0.11	0.03 ± 0.01
Spinnies	North Wales	7.54 ± 0.07	10.61 ± 3.11	13.4 ± 2.3	0.32 ± 0.14	0.09 ± 0.02
Small Heath Park	Birmingham	7.88 ± 0.04	0.42 ± 0.04	5.2 ± 1.3	2.15 ± 1.91	0.39 ± 0.09
Trittiford Mill	Birmingham	7.76 ± 0.05	0.39 ± 0.02	16.2 ± 4.3	1.12 ± 0.88	0.29 ± 0.14
Dudley Donkey Pool	Birmingham	7.96 ± 0.04	0.63 ± 0.02	28.4 ± 22.1	1.07 ± 0.79	0.05 ± 0.02
Whampton West park	Birmingham	7.83 ± 0.07	0.39 ± 0.01	3.2 ± 0.3	4.28 ± 0.78	0.80 ± 0.08
Batley Wilton Park	Leeds	7.75 ± 0.04	0.65 ± 0.01	5.2 ± 0.8	0.86 ± 0.58	0.06 ± 0.01
Bradford Harold Park	Leeds	7.88 ± 0.05	0.38 ± 0.03	5.4 ± 1.7	1.11 ± 0.96	0.04 ± 0.01
Wakefield Calder Park	Leeds	8.06 ± 0.07	0.49 ± 0.02	8.6 ± 5.0	1.01 ± 0.76	0.03 ± 0.01
Birkenhead Park	Liverpool	7.96 ± 0.05	0.49 ± 0.01	2.7 ± 0.3	1.12 ± 0.72	0.07 ± 0.01
Lock Stanley	Liverpool	8.10 ± 0.03	0.45 ± 0.02	1.6 ± 0.3	1.07 ± 0.66	0.06 ± 0.01
Southport Marine Lake	Liverpool	8.22 ± 0.04	14.41 ± 1.75	4.3 ± 0.5	1.01 ± 0.77	0.08 ± 0.01
Alexandra Park	Manchester	8.09 ± 0.08	0.23 ± 0.00	3.5 ± 0.5	0.80 ± 0.58	0.05 ± 0.02
Sale Water Park	Manchester	7.94 ± 0.02	0.58 ± 0.01	1.9 ± 0.1	1.09 ± 0.75	0.05 ± 0.01
Salford Erie Basin	Manchester	8.14 ± 0.03	0.35 ± 0.00	0.7 ± 0.1	0.99 ± 0.75	0.10 ± 0.03
Hanley Park	Stoke on Trent	7.99 ± 0.05	0.36 ± 0.00	1.9 ± 0.2	1.11 ± 0.77	0.05 ± 0.01
Holden Lane pools	Stoke on Trent	8.15 ± 0.05	0.89 ± 0.02	2.2 ± 0.4	1.27 ± 0.78	0.68 ± 0.62

### AMR prevalence

**Figure 3.**
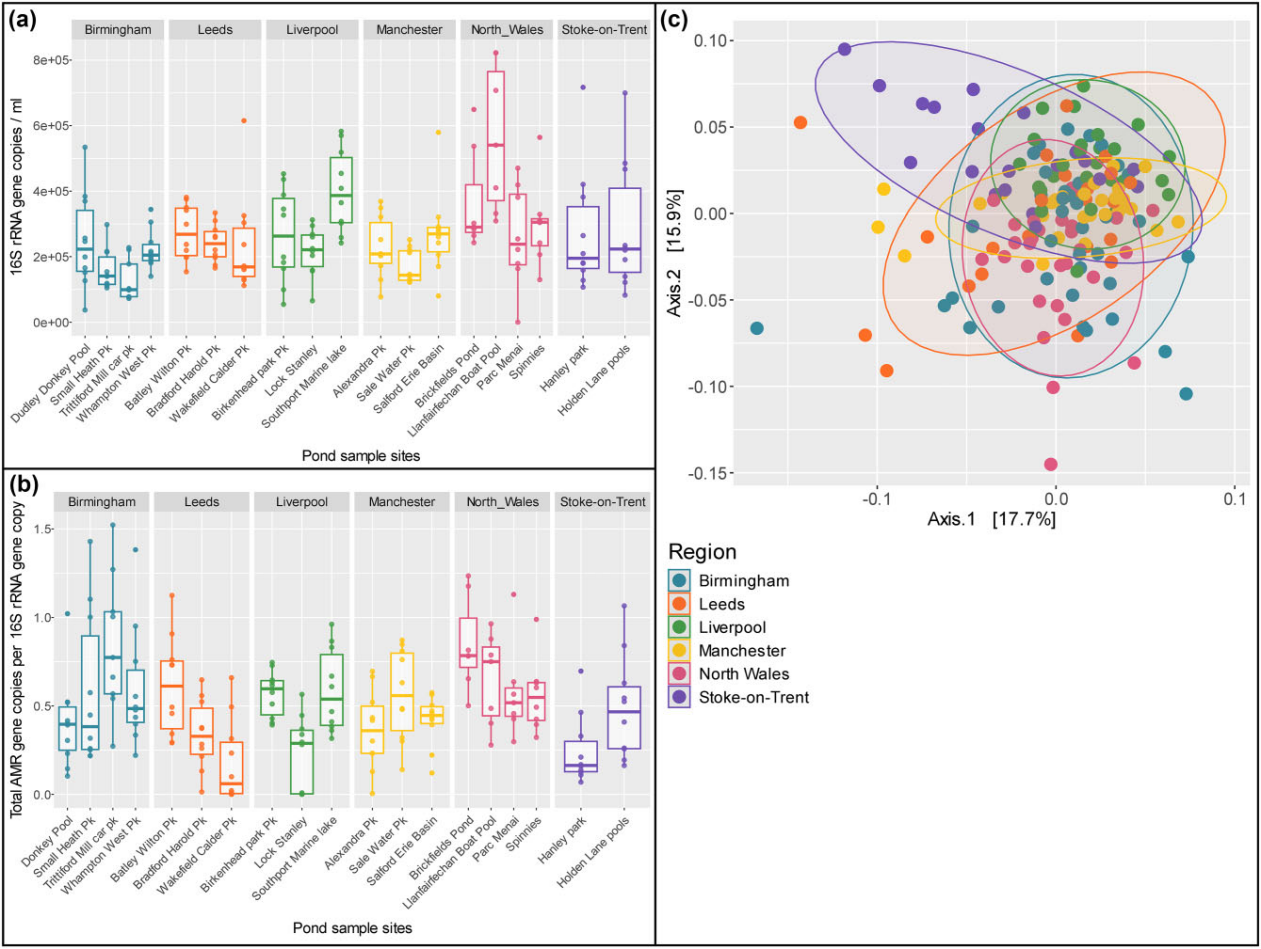
AMR gene profiles in avian-influenced water bodies across different regions of England and Wales. (a) Bacterial abundance measured as 16S rRNA gene copy numbers per ml of water. (b) Relative abundance of antimicrobial resistance genes (ARG) detected in water samples, grouped by geographical region. Data are normalized to 16S rRNA gene copy numbers. (c) Principal coordinates analysis (PCoA) of AMR gene profiles. Individual points represent single samples, with distance between points indicating dissimilarity in resistome composition. Overlapping confidence ellipses (95%) demonstrate similar AMR gene compositions across regions. Temporal analysis showed no significant effect of sampling date on AMR profiles.

Overall, the HT-qPCR data suggested that the total bacteria and AMR profile of sites and regions were similar (Fig. 3). However, despite significant overlap, the composition of the ARG profile did cluster significantly by region (Fig. 3c, PERMANOVA *P* < 0.01), with the strongest dissimilarities observed between Stoke-on-Trent and North Wales (*R*² = 0.20, *F* = 11.5). Significant differences in the total relative abundance ARGs between regions were noted on three occasions (ANOVA and Tukey’s HSD (Honestly Significant Difference), Leeds–Birmingham, *P* = 0.023, Leeds–North Wales, *P* < 0.005, Stoke-on-Trent–North Wales *P* = 0.011), and no differences between sampling sites were observed (*P* > 0.05) (Fig. S1). At most sites, the study period was too short to reveal any seasonal changes in AMR abundance; however, slight temporal variation in the total abundance of AMR genes was observed at Parc Menai and the Spinnies (North Wales) over 10 months, decreasing in the winter (Fig. S1c). The generalized additive model did not find the trends to be statistically significant, although there was some indication of variation at the Spinnies site (*F* = 2.532, *P* = 0.0786, *n* = 53).

**Figure 4.**
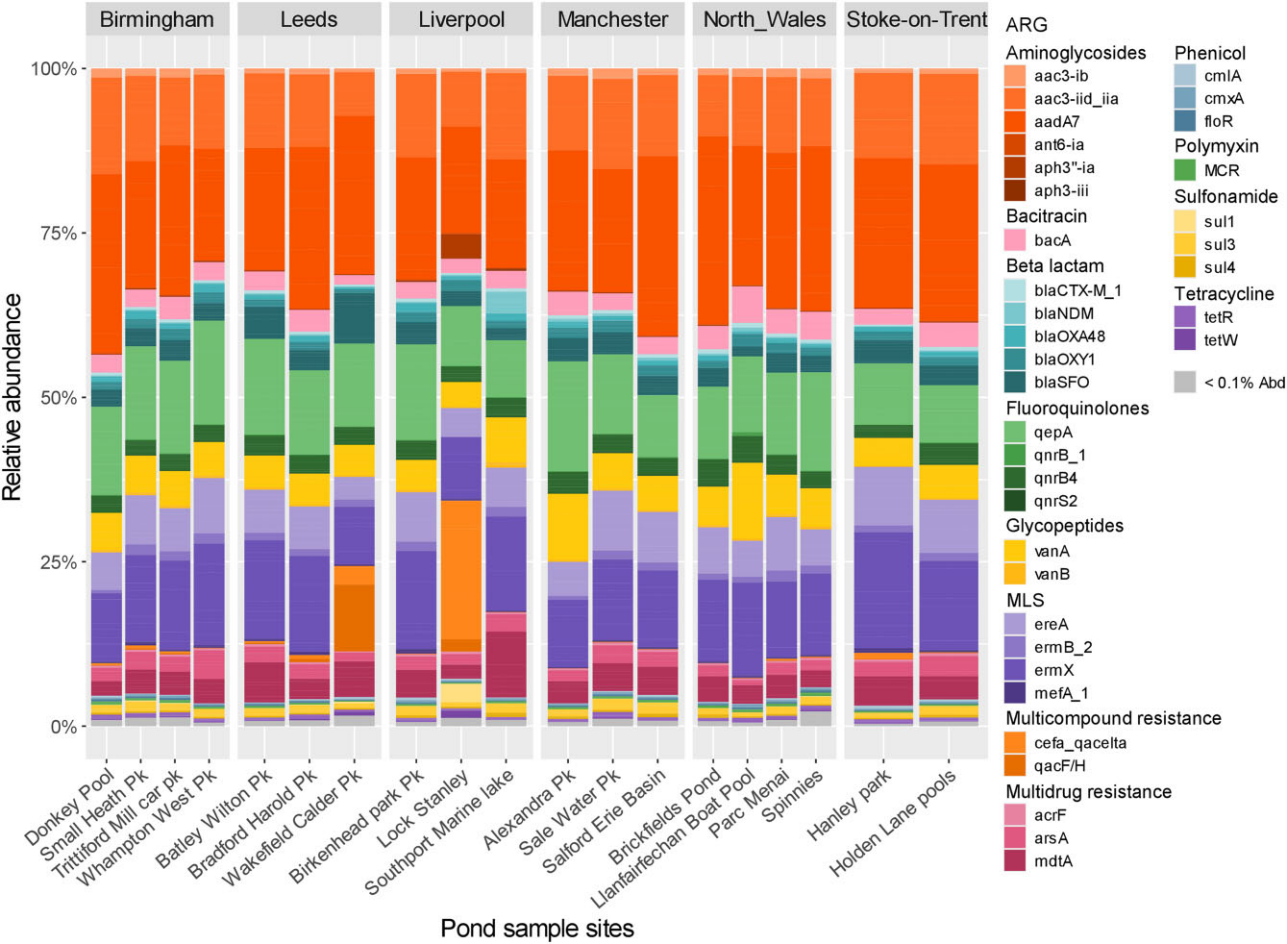
Relative abundance of AMR genes detected in avian-populated water bodies from different regions in England and Wales. Data are grouped by geographical region and presented as normalized abundance values. Colours indicate different AMR gene classes, and bar heights represent mean relative abundance. Genes with abundance of <0.1% are shown in grey.

The AMR gene *aadA7* conferring resistance to aminoglycoside was the most abundant, followed by *ermX* and *qepA* conferring resistance to macrolide-lincosamide-streptogramin B (MLSB) and quinolone, respectively (Fig. 4). AMR genes associated with MDR were noted at lower levels at all sites throughout the study, except for sites Lock Stanley (Liverpool region) and Wakefield Calder Park (Leeds region), where genes *cefa_qacelta* and *qacF/H* were abundant (Fig. 4). The *mcr1* gene, which confers resistance towards the last resort drug, colistin, was detected in 92% of the samples. Our gene panel included four from the ‘big 5’ carbapenemase genes (*bla*_KPC_, *bla*_VIM_, *bla*_NDM_, and *bla*_OXA-48_) (Henderson et al. 2020, Johnson et al. 2024) and all four genes were detected in most samples, with *bla*_OXA-48_ and *bla*_NDM_ exhibiting the highest relative abundance.

Spearman’s correlation analysis revealed a significant and strong positive correlation between the integron encoding *intI3*, and both the high number of ARGs and the total abundance of the resistome, suggesting it could serve as a potential AMR indicator (Fig. S2, Table S4). No significant correlation between the targeted genes and water physico-chemical properties (summarized in Table 1) was observed. The most frequently detected gene associated with pathogens was *Shigella* spp. specific (63% detection rate) (Fig. S3). *Enterococci* spp. and *Staphylococcus aureus* genes were not detected in any of the samples, while *Escherichia coli* and *Candida albicans* genes were only detected in six and two samples, respectively.

### Virus concentration in environmental water samples

The SMF method was tested on seawater, brackish water and freshwater samples in spiking experiments. Across the different samples we found that 1.3% ± 0.6% (range 0.1%–3.3%) of viruses derived from the ATCC stock were recovered using the SMF method. Higher viral recovery rates were noted in freshwater (2.01% ± 1.11%, *n* = 3) and in seawater samples (2.09%, *n* = 1) compared to brackish water (0.15 ± 0.04%, *n* = 2). Sample pH (7.8 ± 0.2) and turbidity (3.8 ± 0.5 NTU) were similar in the samples; only electrical conductivity showed variation (2.6 ± 1.7 mS/cm; Table S1) due to different salinity levels.

### Influenza decay in environmental water samples

**Figure 5.**
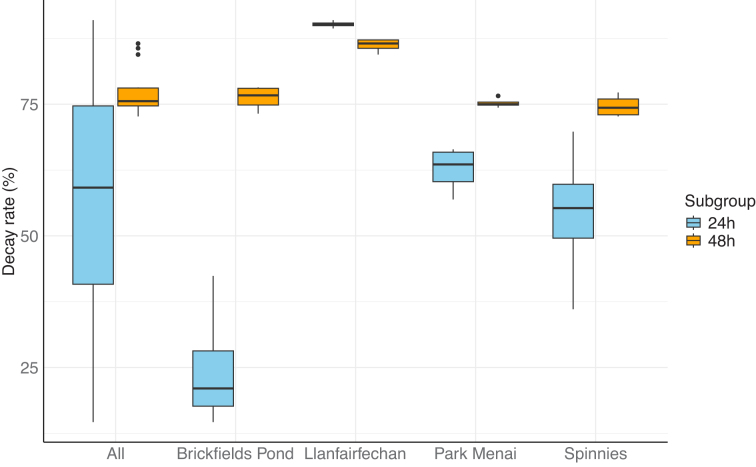
Influenza virus (H3N2) decay rate over 24 h (left-hand blue bars) and 48 h (rigth-hand orange bars) in water samples collected at four locations in North Wales. Sample volume: 3 l, RNA extracted in four replicates.

We explored the degradation of influenza A virus in four water samples over 48 h to assess the effect of sample transport time on viral detection. Overall, viral decay ranged from 25% to 90% after 24 h and reached 75%–86% decay after 48 h (Fig. 5) and the difference was statistically significant (*t*-test, *P* < 0.01, *n* = 16). In one instance, the decay after 24 h was higher than the decay rate observed after 48 h (86% vs. 90%; Fig. 5), which was probably due to slight differences in spiking concentration and/or viral recoveries during process. No significant correlations were observed between viral decay rates and the physico-chemical parameters of water samples.

### Prevalence of influenza viruses in the aquatic environment in England and Wales

Overall, eight samples (3.4%) were positive for influenza A virus during the study period. That included the detection of the virus in North Wales in 2023 on five occasions and detections in North Wales and in the Manchester area on three occasions in 2024 (Table S1). The virus was detected twice at the Brickfields Pond and Spinnies sites (North Wales) and once at Parc Menai and Llanfairfechan sites (North Wales), and at Sale Water Park and Salford Erie Basin (Manchester area). Overall, the concentration of influenza A virus was low with the highest concentrations observed at Sale Water Park (322 gc/l) and the lowest concentration was noted at Salford Erie Basin (22.3 gc/l). The influenza positive samples were negative for H5N1 subtype using RT-PCR.

### Exploring human vs. bird-derived pollution

**Figure 6.**
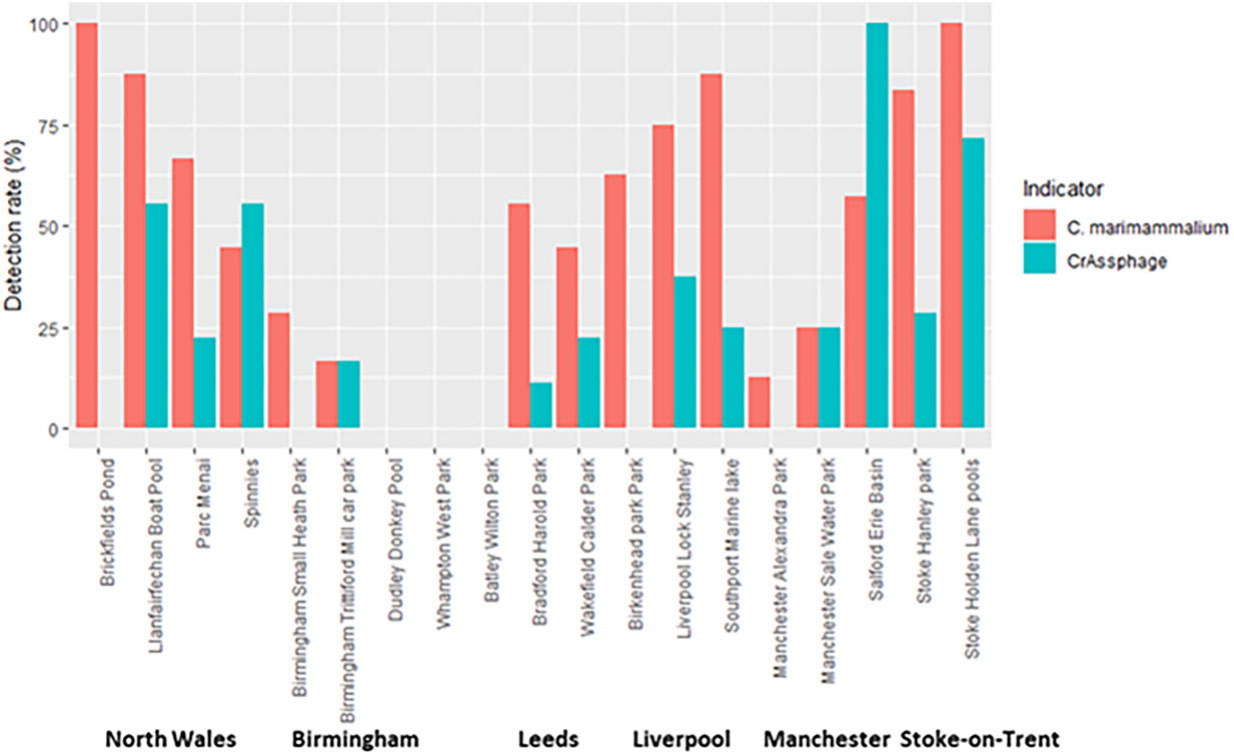
Detection of host-specific faecal markers in water samples across England and Wales. Box plots show the relative abundance of the avian-specific marker *C. marimammalium* (left-hand pink bars) and human-specific marker crAssphage (right-hand blue bars) in water samples collected over a 10-week period. Asterisks (*) indicate sampling sites where influenza A virus was detected.

Overall, 50% of the samples (*n* = 161) tested positive for *C. marimammalium*, while 24% of the samples (*n* = 167) were positive for crAssphage. There were significant differences in the concentration of these indicators among sites (Kruskal–Wallis test, *P* < 0.001). Five sites had negligible *C. marimammalium* prevalence, whereas all the other sites had high abundance of the bacterium. CrAssphage was less frequently detected and was found at seven sites (Fig. 6). At three sites, neither indicator was detected, whereas at four sites only *C. marimammalium* was noted. The remaining sites, including those where influenza virus was detected, had mixed contamination. The *C. marimammalium* and crAssphage showed weak but significant correlation (Spearman's correlation, ρ = 0.34, *P* < 0.001). Weak to moderate positive correlation was observed between *C. marimammalium* concentrations and water pH, conductivity and ammonium levels whereas its concentration showed weak negative correlation with turbidity (Fig. S2). CrAssphage showed similar correlations with pH and conductivity. A consistent negative correlation was also observed between the concentrations of *C. marimammalium* and some of the AMR genes (Stedtfeld et al. 2018); however, the correlations were mostly weak and insignificant (Fig. S2).

## Discussion

### AMR prevalence

In this study, we found a high relative abundance of AMR genes and MGEs in all samples tested using HT-qPCR. The relative abundance of AMR genes was similar between sites; however, some differences between regions were observed. Some little seasonal patterns were also observed; however, these analyses were hindered by the relatively small number of datapoints. Regional and seasonal differences in AMR profiles should be further investigated. Only two out of 19 sites had slightly different AMR profiles with elevated abundance of MDR genes (Fig. 4). According to the last census data (2021), there were no major differences in the level of urbanization, and the human population in general between these sites and those nearby where the MDR genes were not detected as frequently. It is therefore possible that those genes originated from wildlife (e.g. waterbirds) and the sources of these genes should be further investigated.

Overall, the most abundant AMR gene was the aminoglycoside-resistance coding *aadA7*, which was shown to be the most frequently detected AMR gene in a freshwater lagoon in the Baltic region (Gyraitė et al. 2024). Carbapenemases along with MLSB, quinolone and colistin-resistance encoding genes were also highly abundant, as observed previously in freshwater and recreational water bodies (Nappier et al. 2020, Mukherjee et al. 2021, Gyraitė et al. 2024). Of the 10 MGEs quantified, *intl3* was the most abundant and it correlated well with AMR genes, suggesting that it can be a marker for AMR gene abundance in water samples. Previous studies suggested that the *intl1* gene was also suitable as a marker for AMR prevalence and anthropogenic pollution (Gillings et al. 2014, Ma et al. 2017, Bijlsma et al. 2024); however, more studies are needed on the prevalence of other integrons.

Interestingly, a *Shigella* spp. gene was detected frequently in the samples, while genes of other pathogens were sporadically detected or not found. However, no recent studies are available from Europe or North America on *Shigella* detection in surface water, low detection rates were noted in rivers in South Africa and Iran, varying between 2% and 22% (Teklehaimanot et al. 2015, Shahryari et al. 2017, Shahin et al. 2019). These studies use culturing-based quantification, which would exclude dead and viable but not culturable cells, while the qPCR-based approach used in the current study quantifies all bacterial DNA. The low or non-detection of *E. coli* and *Enterococci* spp. may be due to the high limits of detection of HT-qPCR. This method uses only nanolitres of the samples in the reaction mix, and hence it is possible that targets that are present in the samples at relatively low concentrations would not be present in the subsamples and hence would not be detected. The limit of detection and quantification for this method is yet to be investigated.

### Influenza virus prevalence

In this study, we used an eNA-based method for the rapid detection and quantification influenza viruses and other microorganisms in water samples. We found higher recoveries in freshwater and seawater than in brackish water samples, even though sample pH and salinity was adjusted prior to sample concentration. This suggests that chemical properties of the water samples may affect recovery efficiency and hence future studies should focus on the effect of water chemistry on pathogen capture. The SMF method that we used for water concentration has been successfully applied for the concentration of non-enveloped viruses (e.g. polioviruses and adenoviruses) in seawater, with recoveries exceeding 30% (Bitton et al. 1979, Calgua et al. 2008). This method also recovered severe acute respiratory syndrome coronavirus 2 (SARS-CoV-2) and enveloped virus surrogates in wastewater (Tandukar et al. 2024), suggesting that the method is applicable for enveloped viruses. However, enveloped virus recovery in wastewater was generally lower (<10%) than for non-enveloped viruses (Philo et al. 2021, Salvo et al. 2021, Monteiro et al. 2022) and was comparable with the findings of the current study. This suggests that virus type has a major effect on virus recovery efficiency. It is worth noting that the samples with the lowest recovery rates were spiked with a different stock of cultured viruses, which may have influenced recovery efficiency.

An advantage of the SMF method is that it begins with lowering the pH of the sample to 3.5, which inactivates any viable viruses that are present in the sample eliminating the risk of infection. The pH adjustment can be performed in the field as well, if a BSL2/CL2 laboratory is not available for sample processing. Further, this method concentrates viruses along with cellular organisms and so one sample can be used to test different pathogens and microbes for a detailed health risk assessment. The subsequent RNA/DNA extraction method has also been shown to be suitable for the co-extraction of nucleic acids from different pathogens (Loens et al. 2008, Farkas et al. 2022). For influenza virus detection and quantification, we utilized well-established protocols, which have been shown to detect the target sequences accurately and sensitively (WHO 2017, Shu et al. 2021, Farkas et al. 2022).

After establishing an approach for the safe concentration of influenza viruses in water samples, we performed an experiment to better understand the decay of the target virus during sample transit. The results of this persistency experiment suggested less than one log reduction in influenza virus concentrations over 2 days, which is in line with a recent meta-analysis suggesting that AIV remain infectious in water for 0–48 days (Dalziel et al. 2016). It has been suggested that >25% of influenza viruses remained viable in water for up to 7–12 months (Ramey et al. 2022). One study also suggested that viral RNA may be detected even after infectious viruses were not present; however, no quantitative data were provided (Reeves et al. 2020). To date, no studies on the short-term decay of influenza virus RNA in water have been conducted. To limit viral decay in the samples during transport and storage, we recommend that samples collected for influenza RNA studies are transported to the process laboratory on the same day of collection and processed upon arrival, as with this study.

Our results from influenza monitoring in water in North Wales and England suggest that the sampling and sample process approaches we utilized are suitable for the monitoring of influenza viruses in surface water bodies. However, only eight samples were positive for influenza virus in North Wales and in the Manchester area during the study period. Nonetheless, the detection of viral RNA in the Manchester area coincided with the detection of H5N5 highly pathogenic influenza virus in a sparrowhawk (*Accipiter nisus*) (APHA 2025). During the study period, only seven incidents among wild birds in the study area were reported (APHA 2024), suggesting that the virus was not prevalent in wild bird populations, in line with our findings. While no avian influenza cases were registered in North Wales that coincided with viral detection in water, the frequent detection of influenza in water samples collected in North Wales in late November 2023 and early January 2024 coincided with the emergence of human influenza A viruses among the human population. Hence, it is possible that the influenza viruses detected in the aquatic environment were due to pollution by human-derived sewage. Further data collection would be necessary for accurate source tracking.

In previous studies using culturing-based virus enrichment coupled with sequencing, high abundance of the H5N1 influenza virus was noted in a wetland and a lake in Iowa during the ongoing avian flu pandemic in 2022 (Hubbard et al. 2023). Low prevalence of the H5 influenza viruses was also noted in lakes in France in 2009 (Deboosere et al. 2011) and in Cambodia in 2006 (Vong et al. 2008). Low pathogenic H10N7 virus was also found using culturing in surface water in the Netherlands during an epidemic in 2017 (Germeraad et al. 2020) and in China in 2007 (Zhang et al. 2011). Influenza viruses were also successfully cultured in lake water in early studies (Ito et al. 1995). In contrast, the utilization of direct PCR-based detection of influenza viruses without viral enrichment is yet understudied. Using direct RT-qPCR, the H5N1 virus was identified in surface water in Germany and Sweden in 2021–22 (Ahrens et al. 2023). The H1 gene of the influenza A virus was also recovered using PCR in water and ice samples collected in Siberia in 2001 (Zhang et al. 2006). However, these studies were non-quantitative, providing limited information on the abundance of influenza viruses in the environment. In contrast, the method used in our study, provides quantitative data, which can be useful for temporal monitoring of outbreaks.

However, we attempted to subtype the virus in the positive samples without success. As the low viral titres prevented subtyping, more data collected during outbreaks would be necessary to understand the host-specificity and the risks associated with the detection on influenza viruses in water. In the future, sequencing-based approaches should be considered for subtyping. In the studies published so far, sequencing of the influenza strains in water was only successful after culturing-based enrichment of the viruses (Hubbard et al. 2023) or by Sanger sequencing PCR amplicons (Zhang et al. 2006, 2011). With novel technologies emerging, the sequencing of low-concentration RNA may also be successful.

### Prevalence of faecal markers

In this study, we also monitored the prevalence of human (crAssphage) and bird-associated (*C. marimammalium*) faecal markers to assess the sources of contamination at our study sites. CrAssphage is a human gut-associated bacteriophage which has been successfully implemented as an indicator for human wastewater contamination in various aquatic environments globally (García-Aljaro et al. 2017, Stachler et al. 2017, 2018, Ahmed et al. 2018, Ballesté et al. 2019, Farkas et al. 2019, Malla et al. 2019, Ward et al. 2020, Gyawali et al. 2021). However, *C. marimammalium* was first identified in a porpoise and a grey seal (Lawson et al. 2006), it mainly associates with gulls and pigeons and have been shown to be more abundant in gull faeces than *E. coli* (Koskey et al. 2014), supporting our findings. *Catellicoccus marimammalium* has also been found in water samples collected in the coastal environment, suggesting that it is a suitable marker to assess wild bird-related contamination (Lu et al. 2008, Lee et al. 2013, Sinigalliano et al. 2013). Our results on the prevalence of these indicators suggested that most sites were affected by human and/or bird-derived contamination. Only weak correlations between faecal markers and AMR genes were observed and hence more data is necessary to establish to the exact source of AMR in the water environment. The frequent detection of *C. marimammalium* inland suggests that it can be used as an indicator for freshwater environments as well. It is also possible that species other than gulls and pigeons can carry *C. marimammalium* and hence the host range should be further investigated (Sinigalliano et al. 2013). CrAssphage was also frequently detected, especially in the North Wales and Stoke-on-Trent sites, suggesting human wastewater input can be derived from storm overflows or leaking septic tanks. This supports our hypothesis that the influenza A virus detected in water in North Wales was from human origin. In our study, only eight samples were positive for influenza virus, which was not sufficient to conduct correlation analyses with the indicators. The use of both human and animal-associated markers can give a more comprehensive overview on the potential sources of faecal contamination, which can be useful when zoonotic diseases, such as influenza, are monitored at high abundance.

In this study, we investigated the usefulness of molecular approaches for the monitoring of AMR genes, influenza viruses and faecal indicators suitable for environmental source tracking. We found a high abundance of AMR genes in all samples suggesting that AMR contamination with carbapenemases along with aminoglycoside, MLSB, quinolone, and colistin-resistance encoding genes being the most prevalent. We found that the degradation of the influenza virus is greater over 2 days than within 24 h. Therefore, we recommend that samples for such studies are taken, transported, and processed on the same day for the most accurate results and assessment. Using the SMF method, followed by RNA extraction and RT-qPCR, we found a low prevalence of influenza A virus in England and Wales. This was most likely due to the low infectivity rates among wild birds during the study period. However, more research is needed to improve subtyping of influenza viruses in water samples; the environmental monitoring scheme described here could be applied for zoonotic diseases. We also found that *Shigella* spp. and crAssphage can be useful for tracking human-derived contamination, *C. marimammalium* can be utilized for avian originated pollution and the *intl3* gene can be used for AMR tracking in the aquatic environment. This study shows that the simultaneous detection and quantification of these markers is feasible for the continuous monitoring of the targets which can support the current surveillance of zoonotic diseases and contamination source tracking.

## Supplementary Material

qvaf013_Supplemental_Files

## Data Availability

The data that support the findings of this study are available on the supplementary information.
