# Supplementary material for: Monitoring of antimicrobial resistance genes and influenza viruses in avian-populated water bodies
Source: Sustain Microbiol. 2025 Jul 21;2(2):qvaf013. doi: 10.1093/sumbio/qvaf013 (PMC13375323; doi:10.1093/sumbio/qvaf013)
Supplement: Supplementary file 1 [file qvaf013_Supplemental_Files.zip › IAV_AMR_Pond_Supplementary_Table1.pdf]

Table S1 **Metadata containing sampling site names, sampling dates, sample physico-chemical properties, sample process type, influenza virus, crAssphage and Catellicoccus marimammalium concentrations**

Sample ID	Site Name	Sample time	Region	pH	Conductivity (us/cm)	Ammonium (mg/l)	Phosphate (mg/l)	Turbidity (ntu)	Sample process	IAV gc/l	Cmam gc/l	CrAss gc/l	Subject to subtyping by PCR	Subject to subtyping by sequencing
47720	Alexandra Park	16/01/2024	🗺 Manchester	7.88	239.9	0	0	2.4	SMF	0			No	No
47737	Alexandra Park	23/01/2024	🗺 Manchester	7.89	237.8	0.15	0	2.0	SMF	0			No	No
47752	Alexandra Park	30/01/2024	🗺 Manchester	7.94	230.8	1.96	0	2.6	SMF	0	62345.61	0.00	No	No
47779	Alexandra Park	06/02/2024	🗺 Manchester	7.96	221.9	0.3	0	2.0	SMF	0	0.00	0.00	No	No
47798	Alexandra Park	13/02/2024	🗺 Manchester	8.1	234.9	0	0	4.6	SMF	0	0.00	0.00	No	No
47817	Alexandra Park	20/02/2024	🗺 Manchester	8.07	229.2	0	0.05	3.5	SMF	0	0.00	0.00	No	No
47836	Alexandra Park	27/02/2024	🗺 Manchester	8.03	222.8	0	0	3.2	SMF	0	0.00	0.00	No	No
48011	Alexandra Park	05/03/2024	🗺 Manchester	8.06	221.5	0	0	2.6	SMF	0	0.00	0.00	No	No
48026	Alexandra Park	12/03/2024	🗺 Manchester	8.29	228.7	0	0.09	4.7	SMF	0	0.00	0.00	No	No
48041	Alexandra Park	19/03/2024	🗺 Manchester	8.72	227.0	0	0.01	7.0	SMF	0	0.00	0.00	No	No
47728	Batley Wilton Park	17/01/2024	🗺 Leeds	7.64	668.0	0	0	4.8	SMF	0			No	No
47745	Batley Wilton Park	24/01/2024	🗺 Leeds	8.01	652.1	0.15	0	6.3	SMF	0	0.00	0.00	No	No
47760	Batley Wilton Park	31/01/2024	🗺 Leeds	7.78	614.9	2.02	0	9.3	SMF	0	0.00	0.00	No	No
47783	Batley Wilton Park	07/02/2024	🗺 Leeds	7.79	659.6	0.42	0	2.1	SMF	0	0.00	0.00	No	No
47805	Batley Wilton Park	14/02/2024	🗺 Leeds	7.69	635.3	0	0	4.8	SMF	0	0.00	0.00	No	No
47821	Batley Wilton Park	21/02/2024	🗺 Leeds	7.91	581.7	0	0.08	6.4	SMF	0	0.00	0.00	No	No
47840	Batley Wilton Park	28/02/2024	🗺 Leeds	7.65	655.7	0	0	3.9	SMF	0	0.00	0.00	No	No
48015	Batley Wilton Park	06/03/2024	🗺 Leeds	7.64	673.2	0	0	3.1	SMF	0	0.00	0.00	No	No
48030	Batley Wilton Park	13/03/2024	🗺 Leeds	7.66	663.2	0	0.06	2.9	SMF	0	0.00	0.00	No	No
48045	Batley Wilton Park	20/03/2024	🗺 Leeds	7.7	650.9	0	0.04	8.8	SMF	0	0.00	0.00	No	No
47724	Birkenhead Park	17/01/2024	🗺 Liverpool	7.6	469.5	0	0.08	3.5	SMF	0			No	No
47741	Birkenhead Park	24/01/2024	🗺 Liverpool	7.96	492.4	0	0	3.7	SMF	0	0.00	0.00	No	No
47756	Birkenhead Park	31/01/2024	🗺 Liverpool	7.94	484.9	1.84	0.06	3.3	SMF	0			No	No
47786	Birkenhead Park	07/02/2024	🗺 Liverpool	7.98	498.4	0.4	0.05	2.9	SMF	0	0.00	0.00	No	No
47802	Birkenhead Park	14/02/2024	🗺 Liverpool	7.98	508.7	0	0.06	2.6	SMF	0	44441.01	0.00	No	No
47824	Birkenhead Park	21/02/2024	🗺 Liverpool	8.12	491.9	0	0.08	2.1	SMF	0	0.00	0.00	No	No
47843	Birkenhead Park	28/02/2024	🗺 Liverpool	7.85	526.1	0	0	2.2	SMF	0	6847.42	0.00	No	No
48018	Birkenhead Park	06/03/2024	🗺 Liverpool	8.09	391.7	0	0	1.0	SMF	0	7023.80	0.00	No	No
48033	Birkenhead Park	13/03/2024	🗺 Liverpool	8.05	529.4	0	0.11	3.5	SMF	0	22562.24	0.00	No	No
48048	Birkenhead Park	20/03/2024	🗺 Liverpool	8.01	540.9	0	0	2.5	SMF	0	39444.64	0.00	No	No
47727	Bradford Harold Park	17/01/2024	🗺 Leeds	7.57	327.0	0	0	7.3	SMF	0			No	No
47744	Bradford Harold Park	24/01/2024	🗺 Leeds	7.83	331.8	0	0	16.5	SMF	0	0.00	0.00	No	No
47759	Bradford Harold Park	31/01/2024	🗺 Leeds	7.86	553.0	2.07	0	5.7	SMF	0	0.00	0.00	No	No
47782	Bradford Harold Park	07/02/2024	🗺 Leeds	7.98	320.3	0.15	0.02	1.0	SMF	0	18590.87	401.22	No	No
47804	Bradford Harold Park	14/02/2024	🗺 Leeds	8.07	333.2	0	0	1.9	SMF	0	1911.20	0.00	No	No
47820	Bradford Harold Park	21/02/2024	🗺 Leeds	8.02	320.5	0	0.06	2.5	SMF	0	2688.24	0.00	No	No
47839	Bradford Harold Park	28/02/2024	🗺 Leeds	7.77	325.8	0	0	3.3	SMF	0	70972.83	0.00	No	No
48014	Bradford Harold Park	06/03/2024	🗺 Leeds	7.91	335.3	0	0	0.9	SMF	0	6461.34	0.00	No	No
48029	Bradford Harold Park	13/03/2024	🗺 Leeds	7.89	331.2	0	0.05	2.3	SMF	0	0.00	0.00	No	No
48044	Bradford Harold Park	20/03/2024	🗺 Leeds	7.85	582.5	0	0.03	13.0	SMF	0	0.00	0.00	No	No
47211	Brickfields Pond	09/11/2023	North Wales	8.19	470.3	0	0.00	3.4	SMF	0			No	No
47215	Brickfields Pond	23/11/2023	North Wales	8.32	480.0	0	0.00	3.9	SMF	119			Yes	No
47683	Brickfields Pond	07/12/2023	North Wales	8.06	483.1	1	0.00	2.3	SMF	0			No	No
47700	Brickfields Pond	04/01/2024	North Wales	7.95	485.9	0	0.18	2.8	SMF	175			Yes	Yes
47764	Brickfields Pond	18/01/2024	North Wales	8.06	491.2	0.06	0	2.5	SMF	0			No	No
47768	Brickfields Pond	25/01/2024	North Wales	8.08	490.2	0.06	0	2.5	SMF	0	5602.32	0.00	No	No

47772	Brickfields Pond	01/02/2024	North Wales	8.14	486.4	1.8	0	2.7	SMF	0	2688.23	0.00	No	No
47791	Brickfields Pond	08/02/2024	North Wales	8.17	487.8	0.15	0	2.5	SMF	0	7910.99	0.00	No	No
47810	Brickfields Pond	15/02/2024	North Wales	8.34	493.1	0	0	2.2	SMF	0	809.71	0.00	No	No
47829	Brickfields Pond	22/02/2024	North Wales	8.35	483.8	0	0.05	3.5	SMF	0	7614.89	0.00	No	No
47848	Brickfields Pond	29/02/2024	North Wales	8.25	503.9	0	0	3.6	SMF	0	979.39	0.00	No	No
48072	Brickfields Pond	14/03/2024	North Wales	8.17	491.2	0	0.04	2.0	SMF	0	719.13	0.00	No	No
48076	Brickfields Pond	21/03/2024	North Wales	8.15	499.3	0	0.05	2.5	SMF	0	5363.24	0.00	No	No
47717	Dudley Donkey Pool	15/01/2024	🗺 Birmingham	7.73	575.7	0	0	227.0	SMF	0			No	No
47733	Dudley Donkey Pool	22/01/2024	🗺 Birmingham	7.88	564.8	0	0	3.8	SMF	0			No	No
47748	Dudley Donkey Pool	29/01/2024	🗺 Birmingham	8.05	609.1	1.86	0	4.7	SMF	0			No	No
47773	Dudley Donkey Pool	05/02/2024	🗺 Birmingham	8.06	675.7	0.28	0	3.8	SMF	0	0.00	0.00	No	No
47792	Dudley Donkey Pool	12/02/2024	🗺 Birmingham	8.08	598.0	0	0	6.4	SMF	0	0.00	0.00	No	No
47811	Dudley Donkey Pool	19/02/2024	🗺 Birmingham	8.13	570.6	0	0.06	8.9	SMF	0	0.00	0.00	No	No
47830	Dudley Donkey Pool	26/02/2024	🗺 Birmingham	7.95	559.9	0	0	6.8	SMF	0	0.00	0.00	No	No
48005	Dudley Donkey Pool	04/03/2024	🗺 Birmingham	7.94	661.0	0	0	3.9	SMF	0	0.00	0.00	No	No
48020	Dudley Donkey Pool	11/03/2024	🗺 Birmingham	7.91	773.3	0	0.08	2.5	SMF	0	0.00	0.00	No	No
48035	Dudley Donkey Pool	18/03/2024	🗺 Birmingham	7.87	689.6	0	0.01	16.0	SMF	0	0.00	0.00	No	No
47714	Hanley Park	15/01/2024	🗺 Stoke-on-Trent	7.89	364.9	0	0.03	1.3	SMF	0			No	No
47732	Hanley Park	22/01/2024	🗺 Stoke-on-Trent	7.67	357.3	0	0	2.3	SMF	0			No	No
47747	Hanley Park	29/01/2024	🗺 Stoke-on-Trent	7.97	363.2	1.87	0.04	1.5	SMF	0			No	No
47778	Hanley Park	05/02/2024	🗺 Stoke-on-Trent	8	365.0	0.34	0.03	1.4	SMF	0	16681.94	105.17	No	No
47797	Hanley Park	12/02/2024	🗺 Stoke-on-Trent	8.21	358.4	0	0.04	1.7	SMF	0	11936.29	0.00	No	No
47816	Hanley Park	19/02/2024	🗺 Stoke-on-Trent	8.12	351.9	0	0.1	2.4	SMF	0	0.00	0.00	No	No
47835	Hanley Park	26/02/2024	🗺 Stoke-on-Trent	8.01	361.6	0	0	1.6	SMF	0	334379.30	85.75	No	No
48010	Hanley Park	04/03/2024	🗺 Stoke-on-Trent	7.94	360.6	0	0	2.6	SMF	0	1480.35	0.00	No	No
48025	Hanley Park	11/03/2024	🗺 Stoke-on-Trent	7.95	366.8	0	0.07	1.3	SMF	0	1222.06	0.00	No	No
48040	Hanley Park	18/03/2024	🗺 Stoke-on-Trent	8.16	362.4	0	0.04	2.5	SMF	0		0.00	No	No
47713	Holden Lane	15/01/2024	🗺 Stoke-on-Trent	7.99	717.6	0	0	1.2	SMF	0			No	No
47731	Holden Lane	22/01/2024	🗺 Stoke-on-Trent	7.9	941.4	0	0	2.0	SMF	0			No	No
47746	Holden Lane	29/01/2024	🗺 Stoke-on-Trent	8.09	903.9	2.04	0	1.2	SMF	0			No	No
47777	Holden Lane	05/02/2024	🗺 Stoke-on-Trent	8.12	911.4	0.49	0	0.7	SMF	0	7121.88	0.00	No	No
47796	Holden Lane	12/02/2024	🗺 Stoke-on-Trent	8.2	917.9	0	0	1.7	SMF	0	1381.55	96.90	No	No
47815	Holden Lane	19/02/2024	🗺 Stoke-on-Trent	8.33	872.8	0	0.05	2.5	SMF	0	1105.99	1274.74	No	No
47834	Holden Lane	26/02/2024	🗺 Stoke-on-Trent	8.38	889.1	0	1.91	2.7	SMF	0	1290.61	1531.75	No	No
48009	Holden Lane	04/03/2024	🗺 Stoke-on-Trent	8.27	916.1	0	0	2.4	SMF	0	753.34	453.55	No	No
48024	Holden Lane	11/03/2024	🗺 Stoke-on-Trent	8.02	911.2	0	0.08	4.5	SMF	0	9204.81	0.00	No	No
48039	Holden Lane	18/03/2024	🗺 Stoke-on-Trent	8.16	965.8	0	0	3.0	SMF	0		130.38	No	No
43875	Llanfairfechan	29/08/2023	North Wales	7.75	5631.0		0.00	5.3	SMF	0			No	No
46908	Llanfairfechan	13/09/2023	North Wales	7.30	2961.0	0	0.00	11.3	SMF	0			No	No
46911	Llanfairfechan	27/09/2023	North Wales	7.59	403.4	0	0.07	10.7	SMF	0			No	No
47155	Llanfairfechan	11/10/2023	North Wales	7.82	4681.0	0	0.00	9.5	SMF	0			No	No
47159	Llanfairfechan	25/10/2023	North Wales	8.04	4445.0	0	0.07	9.2	SMF	0			No	No
47213	Llanfairfechan	09/11/2023	North Wales	8.03	5827.0	0	0.05	7.8	SMF	0			No	No
47217	Llanfairfechan	23/11/2023	North Wales	7.99	5542.0	0	0.00	8.6	SMF	131			Yes	No
47685	Llanfairfechan	07/12/2023	North Wales	7.70	4841.0	0	0.00	5.9	SMF	0			No	No
47702	Llanfairfechan	04/01/2024	North Wales	7.46	3574.0	0	0.12	4.6	SMF	0			No	No
47762	Llanfairfechan	18/01/2024	North Wales	7.7	3367.0	0	0	4.5	SMF	0			No	No
47766	Llanfairfechan	25/01/2024	North Wales	7.87	3085.0	0.21	0	7.7	SMF	0	116654.40	496.50	No	No
47770	Llanfairfechan	01/02/2024	North Wales	7.35	2840.0	1.92	0	7.0	SMF	0	72964.64	0.00	No	No
47789	Llanfairfechan	08/02/2024	North Wales	7.86	2721.0	0.18	0.03	7.4	SMF	0	14582.03	240.72	No	No

47808	Llanfairfechan	15/02/2024	North Wales	7.78	5370.0	0	0	5.4	SMF	0	30145.41	196.48	No	No
47827	Llanfairfechan	22/02/2024	North Wales	7.72	5232.0	0	0.01	3.8	SMF	0	8057.14	276.90	No	No
47846	Llanfairfechan	29/02/2024	North Wales	8.09	4917.0	0	0	4.8	SMF	0	807.00	0.00	No	No
48066	Llanfairfechan	07/03/2024	North Wales	8.2	4833.0	0	0.1	3.0	SMF	0		490.02	No	No
48070	Llanfairfechan	14/03/2024	North Wales	7.56	7300.0	0	0.04	3.4	SMF	0	59648.65	0.00	No	No
48074	Llanfairfechan	21/03/2024	North Wales	7.74	7402.0	0	0	3.9	SMF	0	0.00	0.00	No	No
47723	Lock Stanley	17/01/2024	🗺 Liverpool	7.89	409.9	0	0	1.3	SMF	0			No	No
47740	Lock Stanley	24/01/2024	🗺 Liverpool	8.15	439.7	0	0	2.2	SMF	0	0.00	0.00	No	No
47755	Lock Stanley	31/01/2024	🗺 Liverpool	8.1	436.5	1.74	0	0.7	SMF	0			No	No
47785	Lock Stanley	07/02/2024	🗺 Liverpool	8.2	424.2	0.39	0	0.9	SMF	0	3875.06	192.22	No	No
47801	Lock Stanley	14/02/2024	🗺 Liverpool	8.2	412.3	0	0.06	0.9	SMF	0	14061.56	176.64	No	No
47823	Lock Stanley	21/02/2024	🗺 Liverpool	7.99	639.0	0	0.03	2.5	SMF	0	4876.29	0.00	No	No
47842	Lock Stanley	28/02/2024	🗺 Liverpool	8.03	411.3	0	0	1.3	SMF	0	2316.44	0.00	No	No
48017	Lock Stanley	06/03/2024	🗺 Liverpool	8.09	393.2	0	0	1.0	SMF	0	6321.88	77.28	No	No
48032	Lock Stanley	13/03/2024	🗺 Liverpool	8.1	431.3	0	0.06	1.2	SMF	0	1278.28	0.00	No	No
48047	Lock Stanley	20/03/2024	🗺 Leeds	8.22	469.8	0	0.1	4.2	SMF	0	0.00	0.00	No	No
43876	Parc Menai	29/03/2023	North Wales	7.89	997.6		0.00	3.8	SMF	0			No	No
43879	Parc Menai	13/04/2023	North Wales	8.01	824.5	0	0.00	6.8	SMF	0			No	No
41525	Parc Menai	26/04/2023	North Wales	8.12	872.1	0	0.03	4.5	SMF	0			No	No
41529	Parc Menai	11/05/2023	North Wales	8.00	822.0	0	0.00	10.6	SMF	0			No	No
42691	Parc Menai	24/05/2023	North Wales	7.99	1681.0	2	0.00	9.1	SMF	0			No	No
42697	Parc Menai	07/06/2023	North Wales	7.91	974.8	0	0.00	5.9	SMF	0			No	No
43863	Parc Menai	28/06/2023	North Wales	8.26	933.6	0	0.00	6.9	SMF	0			No	No
43865	Parc Menai	04/07/2023	North Wales	8.06	827.3	0	0.01	14.7	SMF	0			No	No
46909	Parc Menai	13/09/2023	North Wales	7.66	671.3	0	0.00	2.2	SMF	0			No	No
46913	Parc Menai	27/09/2023	North Wales	8.23	701.2	0	0.03	2.5	SMF	0			No	No
47157	Parc Menai	11/10/2023	North Wales	8.17	724.5	0	0.05	2.5	SMF	0			No	No
47161	Parc Menai	25/10/2023	North Wales	8.22	757.1	0	0.04	1.9	SMF	0			No	No
47214	Parc Menai	09/11/2023	North Wales	8.08	755.0	0	0.00	1.8	SMF	0			No	No
47218	Parc Menai	23/11/2023	North Wales	8.46	799.0	0	0.00	5.7	SMF	117			Yes	No
47686	Parc Menai	07/12/2023	North Wales	7.93	885.5	0	0.00	1.8	SMF	0			No	No
47703	Parc Menai	04/01/2024	North Wales	7.70	664.3	0	0.20	3.8	SMF	0			No	No
47763	Parc Menai	18/01/2024	North Wales	7.94	948.7	0.33	0.04	1.8	SMF	0			No	No
47767	Parc Menai	25/01/2024	North Wales	7.79	881.7	0.06	0.22	4.1	SMF	0	13998.14	199.87	No	No
47771	Parc Menai	01/02/2024	North Wales	7.74	798.6	1.8	0	3.6	SMF	0	935.97	0.00	No	No
47790	Parc Menai	08/02/2024	North Wales	7.82	823.8	0.42	0.04	5.7	SMF	0	2333.71	0.00	No	No
47809	Parc Menai	15/02/2024	North Wales	8.15	866.2	0	0	3.3	SMF	0	6429.22	0.00	No	No
47828	Parc Menai	22/02/2024	North Wales	8.11	752.9	0	0.04	5.0	SMF	0	0.00	0.00	No	No
47847	Parc Menai	29/02/2024	North Wales	7.89	777.8	0	0	7.7	SMF	0	1172.95	0.00	No	No
48067	Parc Menai	07/03/2024	North Wales	7.68	960.9	0	0.06	1.9	SMF	0	0.00	0.00	No	No
48071	Parc Menai	14/03/2024	North Wales	8	697.4	0	0.1	9.1	SMF	0	0.00	978.56	No	No
48075	Parc Menai	21/03/2024	North Wales	7.91	793.0	0	0	1.9	SMF	0	577.80	0.00	No	No
47721	Sale Water Park	16/01/2024	🗺 Manchester	7.86	534.0	0	9.18	0.9	SMF	323			Yes	Yes
47738	Sale Water Park	23/01/2024	🗺 Manchester	7.95	700.5	0	0	2.4	SMF	0			No	No
47753	Sale Water Park	30/01/2024	🗺 Manchester	7.9	571.2	1.84	0	2.2	SMF	0	239280.02	193.97	No	No
47780	Sale Water Park	06/02/2024	🗺 Manchester	7.92	578.4	0.34	0	1.7	SMF	0	0.00	0.00	No	No
47799	Sale Water Park	13/02/2024	🗺 Manchester	7.99	598.6	0	0	2.0	SMF	0	0.00	0.00	No	No
47818	Sale Water Park	20/02/2024	🗺 Manchester	8.01	562.9	0	0.03	2.3	SMF	0	0.00	0.00	No	No
47837	Sale Water Park	27/02/2024	🗺 Manchester	7.92	553.5	0	0	2.1	SMF	0	0.00	0.00	No	No
48012	Sale Water Park	05/03/2024	🗺 Manchester	7.85	594.9	0	0	1.9	SMF	0	0.00	0.00	No	No

48027	Sale Water Park	12/03/2024	🗺 Manchester	7.94	545.4	0	0.06	2.3	SMF	0	0.00	0.00	No	No
48042	Sale Water Park	19/03/2024	🗺 Manchester	8.03	569.2	0	0	1.6	SMF	0	947.47	156.16	No	No
47722	Salford Erie Basin	16/01/2024	🗺 Manchester	7.95	356.2	0	0	0.6	SMF	0			No	No
47739	Salford Erie Basin	23/01/2024	🗺 Manchester	8.07	359.2	0	0	1.5	SMF	0			No	No
47754	Salford Erie Basin	30/01/2024	🗺 Manchester	8.06	348.8	1.74	0	0.4	SMF	22			Yes	Yes
47781	Salford Erie Basin	06/02/2024	🗺 Manchester	8.11	348.1	0.24	0	0.5	SMF	0	565.29	260.37	No	No
47800	Salford Erie Basin	13/02/2024	🗺 Manchester	8.19	353.3	0	0.17	0.8	SMF	0	0.00	105.71	No	No
47819	Salford Erie Basin	20/02/2024	🗺 Manchester	8.24	344.3	0	0.12	0.8	SMF	0	1330.16	367.67	No	No
47838	Salford Erie Basin	27/02/2024	🗺 Manchester	8.23	350.3	0	0	0.6	SMF	0	223.93	282.84	No	No
48013	Salford Erie Basin	05/03/2024	🗺 Manchester	8.21	347.2	0	0	0.6	SMF	0	0.00	361.13	No	No
48028	Salford Erie Basin	12/03/2024	🗺 Manchester	8.22	348.3	0	0.07	0.6	SMF	0	0.00	1717.98	No	No
48043	Salford Erie Basin	19/03/2024	🗺 Manchester	8.12	350.8	0	0.02	0.7	SMF	0	1485.87	320.31	No	No
47719	Small Heath Park	15/01/2024	🗺 Birmingham	7.91	380.8	0	0.31	2.0	SMF	0			No	No
47735	Small Heath Park	22/01/2024	🗺 Birmingham	7.79	409.5	0	0	6.3	SMF	0			No	No
47750	Small Heath Park	29/01/2024	🗺 Birmingham	7.99	426.0	4.06	0.65	1.6	SMF	0			No	No
47774	Small Heath Park	05/02/2024	🗺 Birmingham	8.04	383.2	0.24	0	1.4	SMF	0	209802.81	0.00	No	No
47794	Small Heath Park	12/02/2024	🗺 Birmingham	7.87	418.1	0	0.7	5.1	SMF	0	0.00	0.00	No	No
47813	Small Heath Park	19/02/2024	🗺 Birmingham	7.79	324.8	0	0.55	5.4	SMF	0	0.00	0.00	No	No
47832	Small Heath Park	26/02/2024	🗺 Birmingham	7.97	376.0	0	0	1.8	SMF	0	16575.52	0.00	No	No
48006	Small Heath Park	04/03/2024	🗺 Birmingham	7.7	348.5	0	0.24	12.5	SMF	0	0.00	0.00	No	No
48021	Small Heath Park	11/03/2024	🗺 Birmingham	7.95	763.2	0	0.07	3.9	SMF	0	0.00	0.00	No	No
48036	Small Heath Park	18/03/2024	🗺 Birmingham	7.78	358.6	0	0.24	11.6	SMF	0	0.00	0.00	No	No
47725	Southport Marine lake	17/01/2024	🗺 Liverpool	7.98	8626.0	0	0.07	4.3	SMF	0			No	No
47742	Southport Marine lake	24/01/2024	🗺 Liverpool	8.07	8380.0	0	0	8.0	SMF	0	0.00	0.00	No	No
47757	Southport Marine lake	31/01/2024	🗺 Liverpool	8.11	8004.0	1.78	0.07	3.3	SMF	0			No	No
47787	Southport Marine lake	07/02/2024	🗺 Liverpool	8.21	7942.0	0.24	0.09	5.0	SMF	0	195956.47	0.00	No	No
47803	Southport Marine lake	14/02/2024	🗺 Liverpool	8.4	14590.0	0	0	4.6	SMF	0	58650.54	395.67	No	No
47825	Southport Marine lake	21/02/2024	🗺 Liverpool	8.3	19360.0	0	0.06	3.2	SMF	0	8651.09	0.00	No	No
47844	Southport Marine lake	28/02/2024	🗺 Liverpool	8.39	20200.0	0	0	2.7	SMF	0	9933.94	0.00	No	No
48019	Southport Marine lake	06/03/2024	🗺 Liverpool	8.31	19300.0	0	0	2.5	SMF	0	13639.55	0.00	No	No
48034	Southport Marine lake	13/03/2024	🗺 Liverpool	8.2	19120.0	0	0.11	4.0	SMF	0	112463.78	186.36	No	No
48049	Southport Marine lake	20/03/2024	🗺 Liverpool	8.2	18590.0	0	0.07	5.3	SMF	0	29596.36	0.00	No	No
43874	Spinnies	29/03/2023	North Wales	7.01	1166.0		0.00	7.0	SMF	0			No	No
43878	Spinnies	13/04/2023	North Wales	7.75	344.2	0	0.01	7.8	SMF	0			No	No
41524	Spinnies	26/04/2023	North Wales	7.55	5963.0	0	0.01	3.9	SMF	0			Yes	No
41528	Spinnies	11/05/2023	North Wales	7.89	6514.0	0	0.00	6.4	SMF	0			No	No
42690	Spinnies	24/05/2023	North Wales	7.70	44170.0	3	0.00	10.4	SMF	0			No	No
42696	Spinnies	07/06/2023	North Wales	7.79	42220.0	0	0.00	7.7	SMF	0			No	No
43861	Spinnies	28/06/2023	North Wales	7.97	32080.0	0	0.00	68.6	SMF	0			No	No
43864	Spinnies	04/07/2023	North Wales	7.98	853.2	0	0.03	3.1	SMF	0			No	No
46907	Spinnies	13/09/2023	North Wales	7.16	1161.0	0	0.25	12.3	SMF	0			No	No
46912	Spinnies	27/09/2023	North Wales	7.78	2742.0	0	0.24	5.3	SMF	0			No	No
47156	Spinnies	11/10/2023	North Wales	7.68	8553.0	0	0.42	28.9	SMF	0			No	No
47160	Spinnies	25/10/2023	North Wales	8.29	44810.0	0	0.05	3.5	SMF	0			No	No
47212	Spinnies	09/11/2023	North Wales	7.46	1843.0	0	0.10	7.9	SMF	0			No	No
47216	Spinnies	23/11/2023	North Wales	7.64	13560.0	0	0.00	10.7	SMF	52			Yes	No
47684	Spinnies	07/12/2023	North Wales	7.35	759.4	1	0.00	8.7	SMF	0			No	No
47701	Spinnies	04/01/2024	North Wales	7.13	6644.0	1	0.19	14.0	SMF	51			Yes	Yes
47761	Spinnies	18/01/2024	North Wales	7.32	1509.0	0.27	0	5.8	SMF	0			No	No
47765	Spinnies	25/01/2024	North Wales	7.85	44380.0	0.19	0.36	5.0	SMF	0	10036.32	2450.81	No	No

47769	Spinnies	01/02/2024	North Wales	7.21	759.8	2.32	0	17.5	SMF	0	0.00	0.00	No	No
47788	Spinnies	08/02/2024	North Wales	7.3	515.7	0.27	0.04	10.8	SMF	0	179.74	50.13	No	No
47807	Spinnies	15/02/2024	North Wales	7.45	902.3	0	0.06	7.4	SMF	0	0.00	0.00	No	No
47826	Spinnies	22/02/2024	North Wales	7.7	1562.0	0	0.23	30.4	SMF	0	0.00	0.00	No	No
47845	Spinnies	29/02/2024	North Wales	7.08	1485.0	0	0	18.1	SMF	0	660.35	335.18	No	No
48065	Spinnies	07/03/2024	North Wales	7.75	828.8	0	0.08	5.5	SMF	0	0.00	0.00	No	No
48069	Spinnies	14/03/2024	North Wales	7.2	2363.0	0	0.08	28.9	SMF	0	0.00	639.47	No	No
48073	Spinnies	21/03/2024	North Wales	7.1	8120.0	0	0.09	12.8	SMF	0	500.07	104.52	No	No
47736	Trittiford Mill	22/01/2024	🗺 Birmingham	7.83	477.3	0	0	6.2	SMF	0			No	No
47751	Trittiford Mill	29/01/2024	🗺 Birmingham	7.82	418.8	1.99	0	11.8	SMF	0			No	No
47776	Trittiford Mill	05/02/2024	🗺 Birmingham	7.94	454.6	0.24	0	5.5	SMF	0	0.00	0.00	No	No
47795	Trittiford Mill	12/02/2024	🗺 Birmingham	7.85	450.6	0	0.55	4.6	SMF	0	0.00	0.00	No	No
47814	Trittiford Mill	19/02/2024	🗺 Birmingham	7.78	266.7	0	0.1	32.2	SMF	0	0.00	0.00	No	No
47833	Trittiford Mill	26/02/2024	🗺 Birmingham	7.8	343.9	0	0	22.5	SMF	0	0.00	0.00	No	No
48008	Trittiford Mill	04/03/2024	🗺 Birmingham	7.62	328.1	0	0	23.4	SMF	0	0.00	0.00	No	No
48023	Trittiford Mill	11/03/2024	🗺 Birmingham	7.77	409.8	0	0.51	2.5	SMF	0	12006.78	1010.67	No	No
48038	Trittiford Mill	18/03/2024	🗺 Birmingham	7.47	325.4	0	0.01	36.9	SMF	0		0.00	No	No
47726	Wakefield Calder Park	17/01/2024	🗺 Leeds	7.59	601.3	0	0.02	53.3	SMF	0			No	No
47743	Wakefield Calder Park	24/01/2024	🗺 Leeds	8.1	483.1	0	0	3.2	SMF	0	0.00	0.00	No	No
47758	Wakefield Calder Park	31/01/2024	🗺 Leeds	8	511.2	1.77	0	4.9	SMF	0	6346.44	41555.45	No	No
47784	Wakefield Calder Park	07/02/2024	🗺 Leeds	8.14	457.1	0.25	0	10.7	SMF	0	0.00	0.00	No	No
47806	Wakefield Calder Park	14/02/2024	🗺 Leeds	7.91	422.7	0	0	4.6	SMF	0	0.00	0.00	No	No
47822	Wakefield Calder Park	21/02/2024	🗺 Leeds	8.17	495.5	0	0.04	3.2	SMF	0	0.00	0.00	No	No
47841	Wakefield Calder Park	28/02/2024	🗺 Leeds	8.42	475.4	0	0	3.0	SMF	0	635.21	189.87	No	No
48016	Wakefield Calder Park	06/03/2024	🗺 Leeds	8.2	453.9	0	0	0.9	SMF	0	7153.52	0.00	No	No
48031	Wakefield Calder Park	13/03/2024	🗺 Leeds	7.96	541.9	0	0.06	1.4	SMF	0	0.00	0.00	No	No
48046	Wakefield Calder Park	20/03/2024	🗺 Leeds	8.09	492.6	0	0.01	1.2	SMF	0	639.75	0.00	No	No
47718	Whampton West Park	15/01/2024	🗺 Birmingham	7.66	411.4	0	1.07	3.5	SMF	0			No	No
47734	Whampton West Park	22/01/2024	🗺 Birmingham	7.81	417.1	3.3	1	3.4	SMF	0			No	No
47749	Whampton West Park	29/01/2024	🗺 Birmingham	8.04	406.4	5.82	1.12	2.4	SMF	0			No	No
47775	Whampton West Park	05/02/2024	🗺 Birmingham	8.06	425.0	3.72	1.05	1.7	SMF	0	0.00	0.00	No	No
47793	Whampton West Park	12/02/2024	🗺 Birmingham	8.25	388.6	0	0.83	3.2	SMF	0	0.00	0.00	No	No
47812	Whampton West Park	19/02/2024	🗺 Birmingham	7.8	372.1	0	0.79	3.6	SMF	0	0.00	0.00	No	No
47831	Whampton West Park	26/02/2024	🗺 Birmingham	7.78	371.7	0	0.58	2.1	SMF	0	0.00	0.00	No	No
48007	Whampton West Park	04/03/2024	🗺 Birmingham	7.62	366.0	0	0.53	3.1	SMF	0	0.00	0.00	No	No
48022	Whampton West Park	11/03/2024	🗺 Birmingham	7.64	369.9	0	0.57	3.6	SMF	0	0.00	0.00	No	No
48037	Whampton West Park	18/03/2024	🗺 Birmingham	7.67	370.6	0	0.49	5.6	SMF	0		0.00	No	No
Sample used for spiking	Park Menai	12/12/2024	North Wales	7.94	731	0	0.12	4.5						
Sample used for spiking	Brickfields Pond	09/01/2024	North Wales	8.16	537	0.12	0	2.5						
Sample used for spiking	Spinnies	05/03/2024	North Wales	7.53	1412	0	0.05	4.8						
Sample used for spiking	Llanfairfechan	19/03/2024	North Wales	7.59	7533			3.4						
