# Supplementary material for: Monitoring of antimicrobial resistance genes and influenza viruses in avian-populated water bodies
Source: Sustain Microbiol. 2025 Jul 21;2(2):qvaf013. doi: 10.1093/sumbio/qvaf013 (PMC13375323; doi:10.1093/sumbio/qvaf013)
Supplement: Supplementary file 1 [file qvaf013_Supplemental_Files.zip › IAV_AMR_Pond_Supplementary_Table2.pdf]

Table S2 Summary of sample information for those samples sent for high throughput PCR AMR profiling

Sample ID	Site Name	Sample time
41528	Spinnies	11/05/2023
42690	Spinnies	24/05/2023
42696	Spinnies	07/06/2023
43861	Spinnies	28/06/2023
43863	Parc Menai	28/06/2023
43864	Spinnies	04/07/2023
43865	Parc Menai	04/07/2023
43875	Llanfairfechan Boat Pool	29/08/2023
46907	Spinnies	13/09/2023
46908	Llanfairfechan Boat Pool	13/09/2023
46909	Parc Menai	13/09/2023
46911	Llanfairfechan Boat Pool	27/09/2023
46912	Spinnies	27/09/2023
46913	Parc Menai	27/09/2023
47155	Llanfairfechan Boat Pool	11/10/2023
47156	Spinnies	11/10/2023
47157	Parc Menai	11/10/2023
47159	Llanfairfechan Boat Pool	25/10/2023
47160	Spinnies	25/10/2023
47161	Parc Menai	25/10/2023
47215	Brickfields Pond	23/11/2023
47216	Spinnies	23/11/2023
47217	Llanfairfechan Boat Pool	23/11/2023
47218	Parc Menai	23/11/2023
47713	Stoke Holden Lane pools	15/01/2024
47714	Stoke Hanley park	15/01/2024
47717	Dudley Donkey Pool	15/01/2024
47718	Whampton West Pk	15/01/2024
47719	Bham Small Heath Pk	15/01/2024
47720	Mcr Alexandra Pk	16/01/2024
47721	Mcr Sale Water Pk	16/01/2024
47722	Salford Erie Basin	16/01/2024
47723	Lpool Lock Stanley	17/01/2024
47724	Birkenhead park Pk	17/01/2024
47725	Southport Marine lake	17/01/2024
47726	Wakefield Calder Pk	17/01/2024
47727	Bradford Harold Pk	17/01/2024
47728	Batley Wilton Pk	17/01/2024
47731	Stoke Holden Lane pools	22/01/2024
47732	Stoke Hanley park	22/01/2024
47733	Dudley Donkey Pool	22/01/2024
47734	Whampton West Pk	22/01/2024
47735	Bham Small Heath Pk	22/01/2024
47736	Bham Trittiford Mill car pk	22/01/2024
47737	Mcr Alexandra Pk	23/01/2024
47738	Mcr Sale Water Pk	23/01/2024
47739	Salford Erie Basin	23/01/2024
47740	Lpool Lock Stanley	24/01/2024

47741	Birkenhead park Pk	24/01/2024
47742	Southport Marine lake	24/01/2024
47743	Wakefield Calder Pk	24/01/2024
47744	Bradford Harold Pk	24/01/2024
47745	Batley Wilton Pk	24/01/2024
47746	Stoke Holden Lane pools	29/01/2024
47747	Stoke Hanley park	29/01/2024
47748	Dudley Donkey Pool	29/01/2024
47749	Whampton West Pk	29/01/2024
47750	Bham Small Heath Pk	29/01/2024
47751	Bham Trittiford Mill car pk	29/01/2024
47752	Mcr Alexandra Pk	30/01/2024
47753	Mcr Sale Water Pk	30/01/2024
47754	Salford Erie Basin	30/01/2024
47755	Lpool Lock Stanley	31/01/2024
47756	Birkenhead park Pk	31/01/2024
47757	Southport Marine lake	31/01/2024
47758	Wakefield Calder Pk	31/01/2024
47759	Bradford Harold Pk	31/01/2024
47760	Batley Wilton Pk	31/01/2024
47762	Llanfairfechan Boat Pool	18/01/2024
47763	Parc Menai	18/01/2024
47764	Brickfields Pond	18/01/2024
47765	Spinnies	25/01/2024
47766	Llanfairfechan Boat Pool	25/01/2024
47767	Parc Menai	25/01/2024
47768	Brickfields Pond	25/01/2024
47769	Spinnies	01/02/2024
47770	Llanfairfechan Boat Pool	01/02/2024
47771	Parc Menai	01/02/2024
47772	Brickfields Pond	01/02/2024
47773	Dudley Donkey Pool	05/02/2024
47774	Bham Small Heath Pk	05/02/2024
47775	Whampton West Pk	05/02/2024
47776	Bham Trittiford Mill car pk	05/02/2024
47777	Stoke Holden Lane pools	05/02/2024
47778	Stoke Hanley park	05/02/2024
47779	Mcr Alexandra Pk	06/02/2024
47780	Mcr Sale Water Pk	06/02/2024
47781	Salford Erie Basin	06/02/2024
47782	Bradford Harold Pk	07/02/2024
47783	Batley Wilton Pk	07/02/2024
47784	Wakefield Calder Pk	07/02/2024
47785	Lpool Lock Stanley	07/02/2024
47786	Birkenhead park Pk	07/02/2024
47787	Southport Marine lake	07/02/2024
47788	Spinnies	08/02/2024
47789	Llanfairfechan Boat Pool	08/02/2024
47790	Parc Menai	08/02/2024
47791	Brickfields Pond	08/02/2024

47792	Dudley Donkey Pool	12/02/2024
47793	Whampton West Pk	12/02/2024
47794	Bham Small Heath Pk	12/02/2024
47795	Bham Trittiford Mill car pk	12/02/2024
47796	Stoke Holden Lane pools	12/02/2024
47797	Stoke Hanley park	12/02/2024
47798	Mcr Alexandra Pk	13/02/2024
47799	Mcr Sale Water Pk	13/02/2024
47800	Salford Erie Basin	13/02/2024
47801	Lpool Lock Stanley	14/02/2024
47802	Birkenhead park Pk	14/02/2024
47803	Southport Marine lake	14/02/2024
47804	Bradford Harold Pk	14/02/2024
47805	Batley Wilton Pk	14/02/2024
47806	Wakefield Calder Pk	14/02/2024
47807	Spinnies	15/02/2024
47808	Llanfairfechan Boat Pool	15/02/2024
47809	Parc Menai	15/02/2024
47810	Brickfields Pond	15/02/2024
47811	Dudley Donkey Pool	19/02/2024
47812	Whampton West Pk	19/02/2024
47813	Bham Small Heath Pk	19/02/2024
47814	Bham Trittiford Mill car pk	19/02/2024
47815	Stoke Holden Lane pools	19/02/2024
47816	Stoke Hanley park	19/02/2024
47817	Mcr Alexandra Pk	20/02/2024
47818	Mcr Sale Water Pk	20/02/2024
47819	Salford Erie Basin	20/02/2024
47820	Bradford Harold Pk	21/02/2024
47821	Batley Wilton Pk	21/02/2024
47822	Wakefield Calder Pk	21/02/2024
47823	Lpool Lock Stanley	21/02/2024
47824	Birkenhead park Pk	21/02/2024
47825	Southport Marine lake	21/02/2024
47826	Spinnies	22/02/2024
47827	Llanfairfechan Boat Pool	22/02/2024
47828	Parc Menai	22/02/2024
47829	Brickfields Pond	22/02/2024
47830	Dudley Donkey Pool	26/02/2024
47831	Whampton West Pk	26/02/2024
47832	Bham Small Heath Pk	26/02/2024
47833	Bham Trittiford Mill car pk	26/02/2024
47834	Stoke Holden Lane pools	26/02/2024
47835	Stoke Hanley park	26/02/2024
47836	Mcr Alexandra Pk	27/02/2024
47837	Mcr Sale Water Pk	27/02/2024
47838	Salford Erie Basin	27/02/2024
47839	Bradford Harold Pk	28/02/2024
47840	Batley Wilton Pk	28/02/2024
47841	Wakefield Calder Pk	28/02/2024

47842	Lpool Lock Stanley	28/02/2024
47843	Birkenhead park Pk	28/02/2024
47844	Southport Marine lake	28/02/2024
47845	Spinnies	29/02/2024
47846	Llanfairfechan Boat Pool	29/02/2024
47847	Parc Menai	29/02/2024
47848	Brickfields Pond	29/02/2024
48005	Dudley Donkey Pool	04/03/2024
48006	Bham Small Heath Pk	04/03/2024
48007	Whampton West Pk	04/03/2024
48008	Bham Trittiford Mill car pk	04/03/2024
48009	Stoke Holden Lane pools	04/03/2024
48010	Stoke Hanley park	04/03/2024
48011	Mcr Alexandra Pk	05/03/2024
48012	Mcr Sale Water Pk	05/03/2024
48013	Salford Erie Basin	05/03/2024
48014	Bradford Harold Pk	06/03/2024
48015	Batley Wilton Pk	06/03/2024
48016	Wakefield Calder Pk	06/03/2024
48017	Lpool Lock Stanley	06/03/2024
48018	Birkenhead park Pk	06/03/2024
48019	Southport Marine lake	06/03/2024
48020	Dudley Donkey Pool	11/03/2024
48021	Bham Small Heath Pk	11/03/2024
48022	Whampton West Pk	11/03/2024
48023	Bham Trittiford Mill car pk	11/03/2024
48024	Stoke Holden Lane pools	11/03/2024
48025	Stoke Hanley park	12/03/2024
48026	Mcr Alexandra Pk	12/03/2024
48027	Mcr Sale Water Pk	12/03/2024
48028	Salford Erie Basin	12/03/2024
48029	Bradford Harold Pk	13/03/2024
48030	Batley Wilton Pk	13/03/2024
48031	Wakefield Calder Pk	13/03/2024
48032	Lpool Lock Stanley	13/03/2024
48033	Birkenhead park Pk	13/03/2024
48034	Southport Marine lake	13/03/2024
48035	Dudley Donkey Pool	18/03/2024
48036	Bham Small Heath Pk	18/03/2024
48037	Whampton West Pk	18/03/2024
48038	Bham Trittiford Mill car pk	18/03/2024
48039	Stoke Holden Lane pools	18/03/2024
48040	Stoke Hanley park	18/03/2024
48041	Mcr Alexandra Pk	19/03/2024
48042	Mcr Sale Water Pk	19/03/2024
48043	Salford Erie Basin	19/03/2024
48044	Bradford Harold Pk	20/03/2024
48045	Batley Wilton Pk	20/03/2024
48046	Wakefield Calder Pk	20/03/2024
48047	Lpool Lock Stanley	20/03/2024

48048	Birkenhead park Pk	20/03/2024
48049	Southport Marine lake	20/03/2024
48065	Spinnies	07/03/2024
48066	Llanfairfechan Boat Pool	07/03/2024
48067	Parc Menai	07/03/2024
48069	Spinnies	14/03/2024
