# Supplementary material for: Monitoring of antimicrobial resistance genes and influenza viruses in avian-populated water bodies
Source: Sustain Microbiol. 2025 Jul 21;2(2):qvaf013. doi: 10.1093/sumbio/qvaf013 (PMC13375323; doi:10.1093/sumbio/qvaf013)
Supplement: Supplementary file 1 [file qvaf013_Supplemental_Files.zip › IAV_AMR_Pond_Supplementary_Table3.pdf]

Table S3 **Genes targeted using high throughput PCR AMR profiling**

group	gene	assay
Integrons	intI1_1	AY293
Integrons	intI3	AY500
Integrons	intI1_2	AY289
Integrons	intI2_2	AY294
Aminoglycoside	aadA7	AY412
Aminoglycoside	aac(3)-iid_iia	AY393
Aminoglycoside	aac(3)-ib	AY391
Aminoglycoside	ant6-ia	AY415
Aminoglycoside	aph3-iii	AY420
Aminoglycoside	aadA2_3	AY331
Aminoglycoside	strB	AY24
Aminoglycoside	aph(3'')-ia	AY418
Aminoglycoside	aac6-aph2	AY406
Aminoglycoside	aacA/aphD	AY4
Quinolone	qepA	AY456
Quinolone	qnrB4	AY457
Quinolone	qnrB	AY96
Quinolone	qnrS2	AY461
Quinolone	qnrB_2	AY458
Quinolone	qnrA	AY95
MLSB	ermX_2	AY546
MLSB	ereA	AY528
MLSB	ermB_3	AY547
MLSB	mefA	AY538
MLSB	mefA_1	AY65
MLSB	ermA	AY532
MLSB	msrE	AY553
MLSB	lnuB	AY536
MLSB	ermB_1	AY56
Vancomycin	vanA	AY595
Vancomycin	vanB_1	AY159
Vancomycin	vanSA	AY165
Vancomycin	vanRA_1	AY164
MDR	mdtA	AY485
MDR	arsA	AY490
MDR	acrF	AY201
MDR	cefa_qacelta	AY487
MDR	qacF/H	AY489
MDR	tcrB	AY498
Other	bacA	AY465
Other	mcr1	AY466
Other	merA	AY191
Other	qacEâˆ†1_3	AY236
MGE	IS21-ISAs29	AY510
MGE	ISPps	AY309
MGE	ISEcp1	AY520
MGE	tnpA_7	AY305
MGE	tnpA_6	AY304

MGE	tnpA_1	AY299
Beta-Lactam	blaSFO	AY126
Beta-Lactam	blaOXY1	AY436
Beta-Lactam	blaOXA48	AY601
Beta-Lactam	blaCTX-M	AY432
Beta-Lactam	blaNDM	AY152
Beta-Lactam	blaCARB	AY446
Beta-Lactam	blaSHV11	AY438
Beta-Lactam	blaCTX-M_5	AY134
Beta-Lactam	blaVIM	AY129
Beta-Lactam	blaKPC_2	AY153
Beta-Lactam	blaKPC	AY440
Beta-Lactam	blaTEM	AY439
Beta-Lactam	blaOXA58	AY145
Beta-Lactam	blaCTX-M_8	AY147
Beta-Lactam	blaGES	AY125
Beta-Lactam	blaVEB	AY105
Sulfonamide	sul3_1	AY244
Sulfonamide	sul4	AY241
Sulfonamide	sul1_2	AY245
Sulfonamide	sul2_2	AY365
Taxonomic	Shigella	AY625
Taxonomic	Enterococci	AY476
Taxonomic	E.coli-uidA_2	AY631
Taxonomic	C. albicans	AY629
Taxonomic	S. aureus	AY628
Phenicol	cmxA	AY37
Phenicol	floR_1	AY32
Phenicol	cmlA_2	AY35
Phenicol	catB3	AY29
Phenicol	mdtL	AY34
Tetracycline	tetR	AY577
Tetracycline	tetW	AY263
Tetracycline	tetM	AY574
Tetracycline	tetE	AY273
Tetracycline	tetQ	AY259
Tetracycline	tet39	AY568
Trimethoprim	dfrA1_1	AY284
Trimethoprim	dfrA15	AY580
Trimethoprim	dfra17	AY581
Trimethoprim	dfrAB4	AY590
Trimethoprim	dfrG	AY593
16S rRNA	16S rRNA	AY1
