# Supplementary material for: Monitoring of antimicrobial resistance genes and influenza viruses in avian-populated water bodies
Source: Sustain Microbiol. 2025 Jul 21;2(2):qvaf013. doi: 10.1093/sumbio/qvaf013 (PMC13375323; doi:10.1093/sumbio/qvaf013)
Supplement: Supplementary file 1 [file qvaf013_Supplemental_Files.zip › IAV_AMR_Pond_Supplementary_Table4.pdf]

	tcrB	intI1_1	intI1_2	intI3	merA	IS21-ISAs29	ISECP1	ISPPS	tnpA_1	tnpA_2	tnpA_3	Shigella	pH	EC	Ammonia	Phosphate	Turbidity	Cmams	Total
aac3-ib	0.48	0.49	-0.07	0.79*	0.40	0.87 *	0.63	-0.07	0.42	0.21	0.47	0.61	-0.25	0.09	-0.10	0.06	0.34	-0.08	0.85 *
aac3-iid_iia	0.42	0.50	-0.07	0.81*	0.40	0.87 *	0.71 *	-0.10	0.34	0.16	0.28	0.64	-0.14	0.12	-0.14	0.13	0.15	-0.01	0.91 *
aadA2	0.60	0.43	0.18	0.64	0.46	0.69	0.70	0.03	0.50	0.43	0.63	0.49	-0.32	0.02	-0.05	0.10	0.24	-0.20	0.66
aadA7	0.45	0.49	-0.07	0.85*	0.42	0.88 *	0.65	-0.10	0.39	0.16	0.42	0.63	-0.17	0.09	-0.11	0.00	0.31	-0.08	0.93 *
ant6-ia	0.43	0.49	-0.06	0.78*	0.37	0.81 *	0.63	-0.07	0.39	0.18	0.37	0.71 *	-0.02	0.08	-0.02	0.02	0.17	0.00	0.83 *
aph3''-ia	0.29	0.11	0.14	0.15	0.08	0.21	0.26	0.04	0.26	0.13	0.21	0.18	-0.25	0.03	-0.08	-0.14	0.10	-0.04	0.16
aph3-iii	0.38	0.34	-0.15	0.65	0.36	0.76 *	0.65	-0.14	0.25	0.20	0.24	0.60	-0.03	0.09	-0.12	0.15	0.22	0.01	0.77 *
strB	0.48	0.22	0.10	0.50	0.45	0.60	0.51	0.01	0.47	0.42	0.54	0.57	-0.35	0.21	-0.06	-0.01	0.46	-0.23	0.57
bacA	0.40	0.47	-0.18	0.85*	0.37	0.85 *	0.67	-0.19	0.35	0.19	0.33	0.62	-0.16	0.17	0.00	0.03	0.29	0.02	0.89 *
blaCARB	0.41	0.44	-0.15	0.75*	0.28	0.81 *	0.61	-0.16	0.40	0.07	0.31	0.70 *	-0.02	0.11	-0.15	0.15	0.16	0.01	0.84 *
blaCTX-M_1	0.45	0.49	-0.15	0.85*	0.38	0.89 *	0.67	-0.13	0.36	0.12	0.40	0.70	-0.06	0.15	-0.13	0.02	0.26	0.06	0.91 *
blaCTX-M_2	0.36	0.37	-0.09	0.69	0.35	0.76 *	0.59	-0.14	0.33	0.21	0.34	0.70	-0.07	0.15	-0.06	0.02	0.26	-0.07	0.79 *
blaKPC_1	0.38	0.39	-0.07	0.66	0.29	0.70	0.60	-0.01	0.28	0.09	0.30	0.56	0.03	-0.04	-0.12	0.08	0.05	-0.01	0.70
blaKPC_2	0.38	0.47	0.09	0.73*	0.27	0.73 *	0.58	0.18	0.39	0.08	0.43	0.60	0.02	0.04	-0.12	-0.05	0.06	-0.07	0.74 *
blaNDM	0.33	0.41	-0.18	0.72*	0.18	0.70	0.52	-0.13	0.31	0.06	0.26	0.57	0.07	0.08	-0.09	0.04	0.08	0.09	0.77 *
blaOXA48	0.34	0.41	-0.16	0.70	0.30	0.76 *	0.59	-0.17	0.21	0.09	0.21	0.67	0.00	0.00	-0.13	0.14	0.08	0.02	0.81 *
blaOXY1	0.45	0.58	0.03	0.84*	0.41	0.84 *	0.71 *	-0.01	0.36	0.20	0.30	0.58	-0.16	-0.01	-0.09	0.07	0.13	-0.09	0.88 *
blaSFO	0.45	0.51	0.13	0.86*	0.55	0.81 *	0.74 *	0.01	0.32	0.34	0.42	0.55	-0.20	-0.02	-0.13	0.03	0.20	-0.22	0.85 *
blaSHV11	0.47	0.37	-0.02	0.73*	0.48	0.84 *	0.75 *	-0.09	0.34	0.31	0.46	0.61	-0.25	0.30	-0.13	0.01	0.39	-0.09	0.82 *
blaTEM	0.28	0.19	0.08	0.24	0.28	0.38	0.40	0.07	0.30	0.42	0.27	0.16	-0.30	-0.09	-0.02	0.04	0.22	-0.11	0.33
blaVIM	0.38	0.33	0.04	0.58	0.36	0.67	0.48	0.05	0.32	0.08	0.37	0.53	-0.07	0.13	-0.16	-0.01	0.22	-0.03	0.67
cefa_qacelta	0.25	0.06	0.54	-0.02	0.36	0.06	0.26	0.37	0.27	0.31	0.25	0.02	-0.40	0.03	0.04	0.03	0.16	-0.26	-0.05
qacF/H	0.45	0.52	0.15	0.72*	0.56	0.73 *	0.70 *	0.12	0.40	0.31	0.44	0.50	-0.28	0.13	-0.07	0.05	0.22	-0.06	0.71 *
ereA	0.45	0.53	-0.01	0.84*	0.41	0.90 *	0.75 *	-0.08	0.30	0.22	0.33	0.65	-0.14	0.04	-0.13	0.13	0.10	-0.04	0.93 *
ermA	0.43	0.50	-0.08	0.73*	0.34	0.77 *	0.67	-0.12	0.32	0.22	0.34	0.59	-0.15	0.09	-0.09	0.07	0.23	-0.02	0.79 *
ermB_1	0.35	0.04	0.21	0.03	0.33	0.17	0.33	0.14	0.46	0.37	0.42	0.05	-0.39	0.02	0.02	0.00	0.34	-0.16	0.09
ermB_2	0.38	0.41	0.12	0.59	0.42	0.60	0.59	0.12	0.47	0.14	0.42	0.46	-0.17	0.06	-0.22	0.02	0.25	-0.07	0.59
ermX	0.47	0.46	-0.10	0.86*	0.46	0.89 *	0.77 *	-0.23	0.28	0.27	0.30	0.64	-0.24	0.11	-0.11	0.10	0.26	-0.05	0.95 *
mefA_1	0.39	0.38	-0.10	0.65	0.37	0.73 *	0.66	-0.24	0.17	0.10	0.19	0.46	-0.17	-0.03	-0.20	0.19	0.05	0.09	0.75 *
mefA_2	0.51	0.35	0.15	0.51	0.40	0.62	0.59	0.03	0.39	0.39	0.60	0.45	-0.21	0.07	-0.07	0.09	0.15	-0.05	0.56

msrE	0.36	0.15	0.24	0.12	0.28	0.21	0.32	0.29	0.49	0.41	0.46	0.13	-0.34	-0.05	0.05	0.01	0.32	-0.13	0.15
acrF	0.51	0.51	0.06	0.85*	0.48	0.86 *	0.76 *	0.00	0.36	0.22	0.42	0.64	-0.09	0.06	-0.15	0.03	0.15	-0.03	0.86 *
arsA	0.40	0.40	-0.03	0.69	0.36	0.77 *	0.66	-0.13	0.19	0.26	0.27	0.62	-0.10	0.04	-0.06	0.20	0.08	-0.11	0.79 *
mdtA	0.34	0.43	-0.14	0.75*	0.39	0.81 *	0.67	-0.21	0.25	0.24	0.30	0.60	-0.04	0.15	-0.10	0.10	0.15	-0.02	0.87 *
mdtL	0.24	0.21	0.23	0.32	0.22	0.30	0.33	0.31	0.21	0.22	0.27	0.20	-0.07	0.07	-0.12	-0.03	-0.01	-0.14	0.31
catB3	0.39	0.51	0.22	0.51	0.20	0.52	0.31	0.29	0.31	0.10	0.49	0.44	0.03	-0.02	-0.11	-0.06	0.00	-0.04	0.48
cmlA	0.28	0.43	0.18	0.56	0.33	0.55	0.45	0.27	0.16	-0.06	0.19	0.44	0.14	-0.01	-0.22	-0.01	-0.09	0.03	0.56
cmxA	0.50	0.47	-0.09	0.83*	0.47	0.89 *	0.78 *	-0.08	0.32	0.22	0.34	0.64	-0.11	0.17	-0.11	0.09	0.20	0.02	0.87 *
floR	0.50	0.51	0.04	0.79*	0.47	0.84 *	0.66	-0.03	0.49	0.33	0.51	0.61	-0.34	0.06	-0.10	0.01	0.35	-0.17	0.84 *
MCR	0.47	0.51	-0.12	0.82*	0.40	0.91 *	0.70 *	-0.15	0.46	0.19	0.44	0.71 *	-0.18	0.13	-0.13	0.10	0.31	-0.03	0.90 *
qepA	0.50	0.46	-0.04	0.82*	0.56	0.91 *	0.75 *	-0.18	0.37	0.38	0.41	0.60	-0.36	0.07	-0.04	0.11	0.37	-0.15	0.92 *
qnrA	0.25	0.21	0.02	0.29	0.16	0.32	0.27	0.03	0.16	0.09	0.22	0.26	0.03	-0.02	-0.07	-0.03	0.01	-0.02	0.33
qnrB_1	0.46	0.46	-0.13	0.80*	0.36	0.84 *	0.65	-0.11	0.39	0.12	0.38	0.56	-0.13	0.19	-0.08	0.00	0.24	0.05	0.86 *
qnrB_2	0.33	0.42	-0.02	0.71*	0.27	0.73 *	0.53	0.02	0.28	0.13	0.42	0.65	0.05	0.14	-0.07	-0.05	0.18	-0.03	0.76 *
qnrB4	0.39	0.51	-0.18	0.85*	0.38	0.86 *	0.65	-0.17	0.31	0.14	0.35	0.66	-0.07	0.14	-0.06	0.01	0.22	0.06	0.89 *
qnrS2	0.39	0.49	-0.04	0.78*	0.28	0.81 *	0.67	-0.04	0.29	0.12	0.34	0.59	-0.04	-0.01	-0.15	0.11	0.00	0.04	0.82 *
sul1	0.52	0.48	0.21	0.60	0.45	0.62	0.57	0.09	0.48	0.28	0.53	0.51	-0.20	0.17	-0.01	-0.13	0.31	-0.07	0.60
sul3	0.44	0.42	-0.02	0.75*	0.39	0.85 *	0.70 *	-0.08	0.29	0.21	0.38	0.63	-0.06	0.03	-0.15	0.15	0.06	-0.06	0.86 *
sul4	0.33	0.42	-0.18	0.76*	0.27	0.78 *	0.60	-0.10	0.30	0.03	0.30	0.70 *	0.17	0.11	-0.10	0.01	0.09	0.10	0.83 *
tet39	0.29	0.06	0.18	0.17	0.29	0.21	0.41	0.19	0.38	0.30	0.37	0.10	-0.26	0.06	0.11	0.03	0.25	-0.06	0.17
tetE	0.46	0.43	-0.11	0.77*	0.36	0.80 *	0.66	-0.08	0.47	0.13	0.38	0.64	-0.02	0.02	-0.13	0.05	0.20	0.02	0.79 *
tetM	0.52	0.38	0.06	0.65	0.50	0.74 *	0.73 *	-0.02	0.37	0.35	0.46	0.59	-0.23	0.16	-0.10	0.06	0.34	-0.06	0.72 *
tetQ	0.32	0.20	0.28	0.17	0.43	0.30	0.40	0.24	0.52	0.54	0.54	0.14	-0.40	0.11	0.09	-0.03	0.42	-0.26	0.22
tetR	0.52	0.55	-0.01	0.85*	0.44	0.89 *	0.74 *	-0.07	0.36	0.17	0.37	0.68	-0.18	0.08	-0.14	0.09	0.21	-0.04	0.90 *
tetW	0.42	0.27	0.34	0.31	0.51	0.41	0.51	0.20	0.36	0.57	0.52	0.27	-0.48	0.10	0.04	0.03	0.30	-0.30	0.33
dfrA1	0.45	0.48	-0.05	0.78*	0.42	0.82 *	0.73 *	-0.11	0.34	0.22	0.38	0.64	-0.14	0.16	-0.13	0.12	0.22	-0.03	0.83 *
dfrA15	0.44	0.41	0.14	0.64	0.38	0.66	0.55	0.07	0.22	0.23	0.42	0.50	-0.07	0.13	-0.05	-0.07	0.03	-0.06	0.65
dfrA17	0.33	0.22	0.37	0.25	0.32	0.30	0.39	0.19	0.32	0.53	0.46	0.19	-0.30	-0.01	0.06	-0.01	0.14	-0.27	0.26
vanA	0.43	0.46	-0.23	0.81*	0.31	0.88 *	0.65	-0.21	0.34	0.12	0.34	0.66	-0.13	0.20	-0.15	0.07	0.30	0.09	0.90 *
vanB	0.40	0.31	-0.22	0.67	0.41	0.83 *	0.63	-0.29	0.34	0.28	0.38	0.68	-0.20	0.30	-0.03	0.13	0.42	-0.03	0.83 *
ARG_Total	0.48	0.51	-0.11	0.90*	0.46	0.95 *	0.76 *	-0.17	0.36	0.23	0.38	0.69	-0.17	0.12	-0.12	0.10	0.27	-0.05	1.00
