# Supplementary material for: Monitoring of antimicrobial resistance genes and influenza viruses in avian-populated water bodies
Source: Sustain Microbiol. 2025 Jul 21;2(2):qvaf013. doi: 10.1093/sumbio/qvaf013 (PMC13375323; doi:10.1093/sumbio/qvaf013)

Figure S 1 A 16S rRNA gene copy numbers

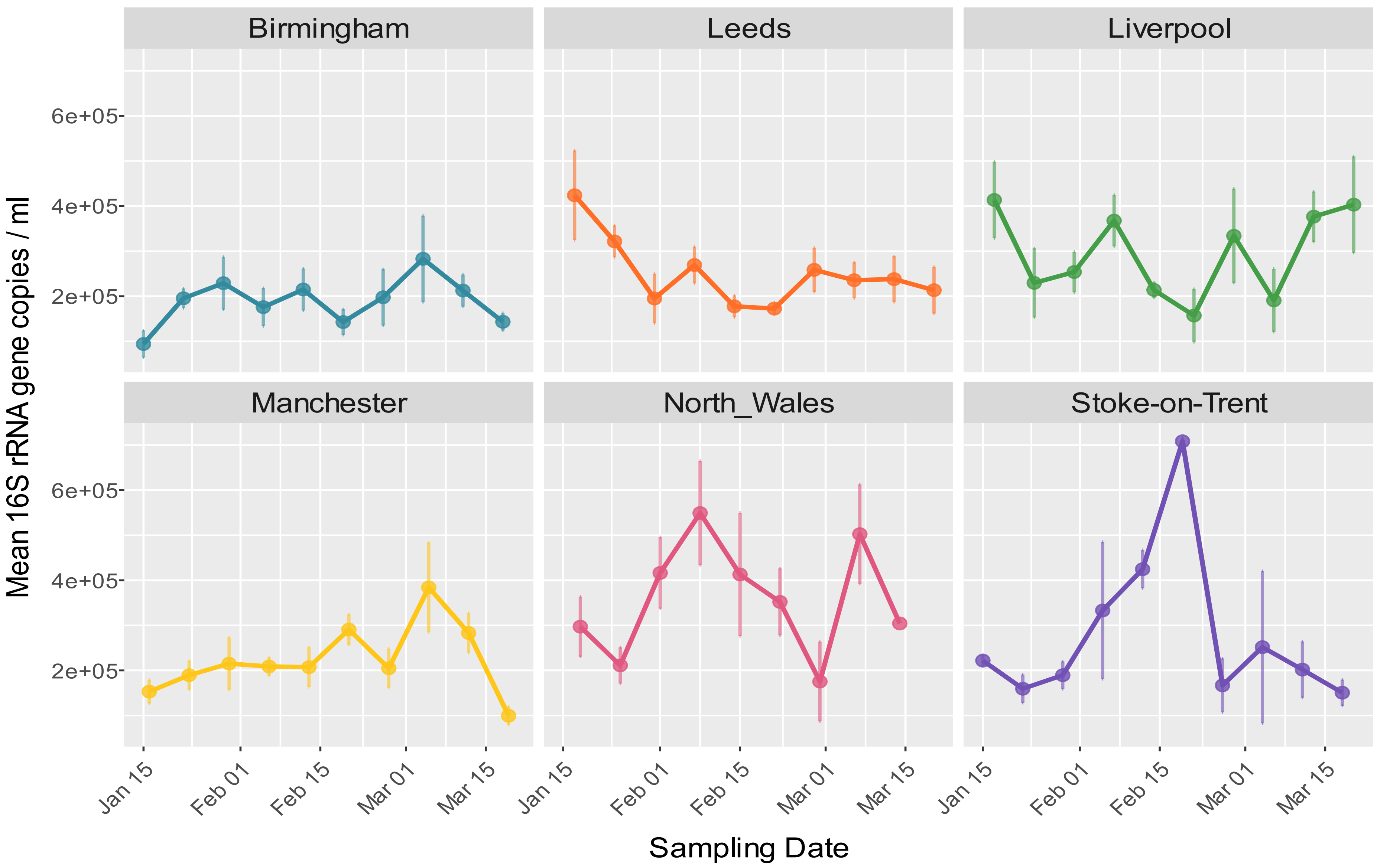

Figure S 1 B Relative abundance of total ARGs detected at each region during the study period

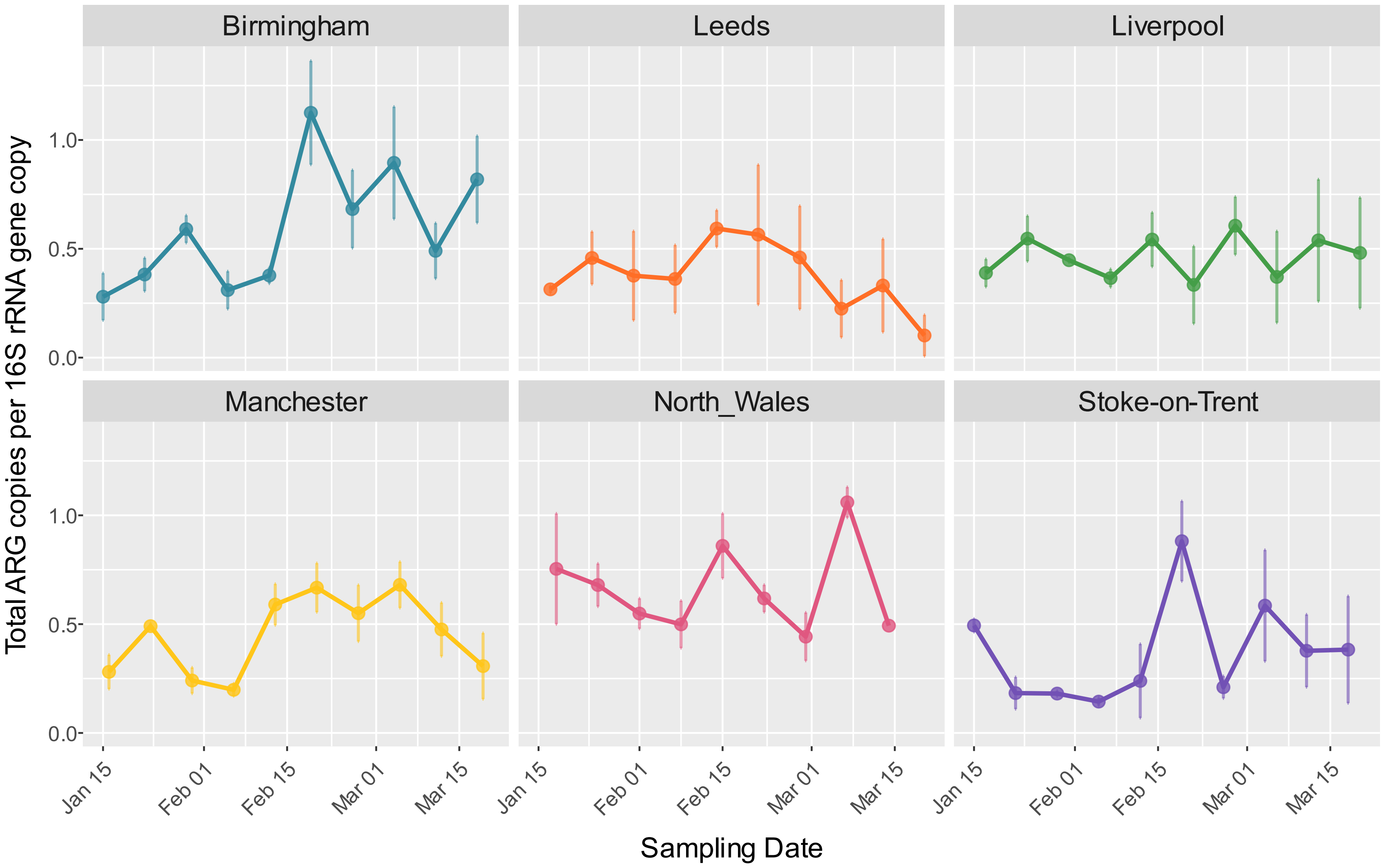

Figure S 1 C Relative abundance of total ARGs detected at North Wales during the study period

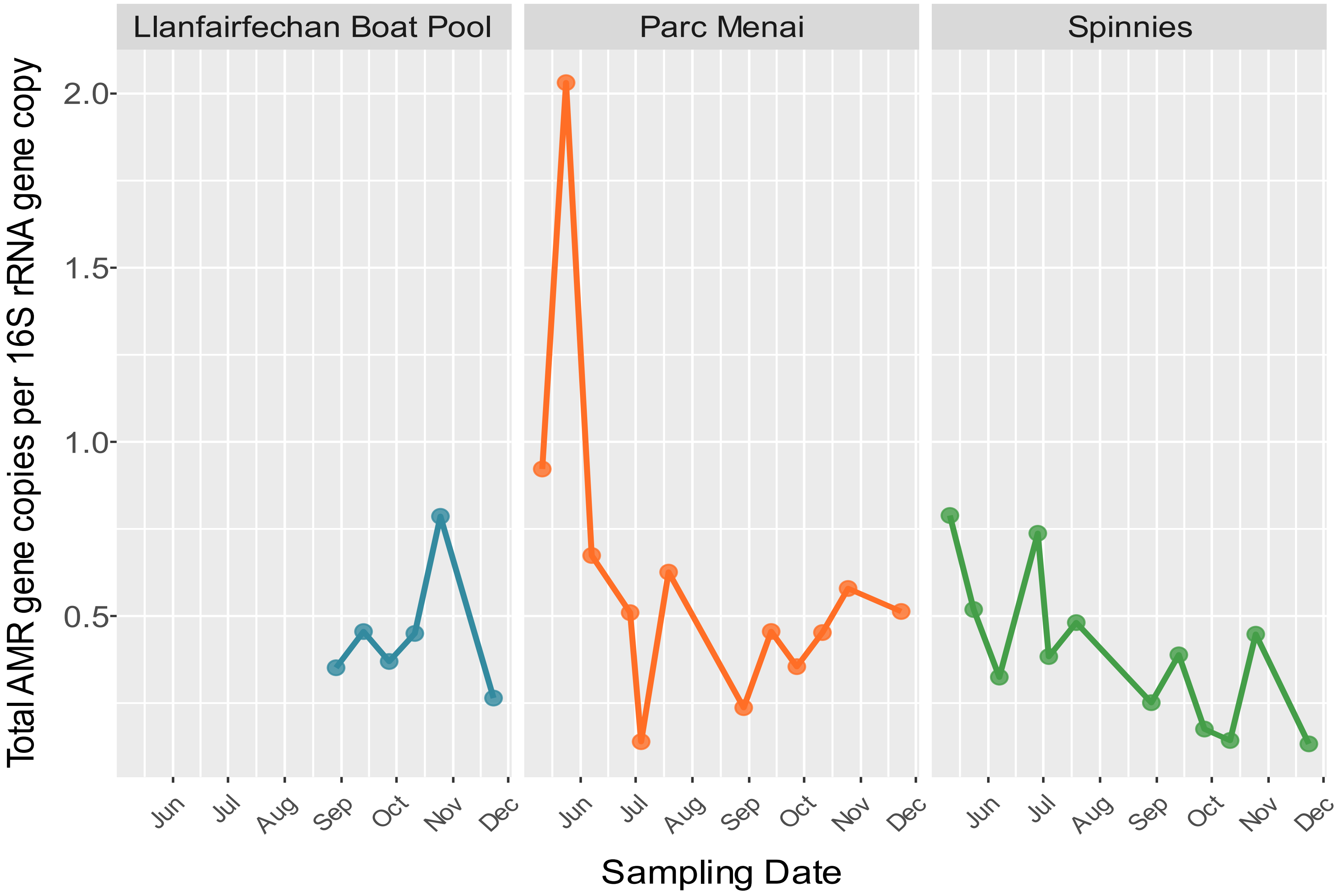

Supplement: Supplementary file 1 [file qvaf013_Supplemental_Files.zip › IAV_AMR_Pond_Supplementary_V4_Figure1.pdf]
