# Supplementary material for: Monitoring of antimicrobial resistance genes and influenza viruses in avian-populated water bodies
Source: Sustain Microbiol. 2025 Jul 21;2(2):qvaf013. doi: 10.1093/sumbio/qvaf013 (PMC13375323; doi:10.1093/sumbio/qvaf013)

Figure S2 Heatmap showing correlations between AMR genes, Shigella, Catellicoccus marimammalium (Cmams), crAssphage (CrAss) and water physico-chemical properties

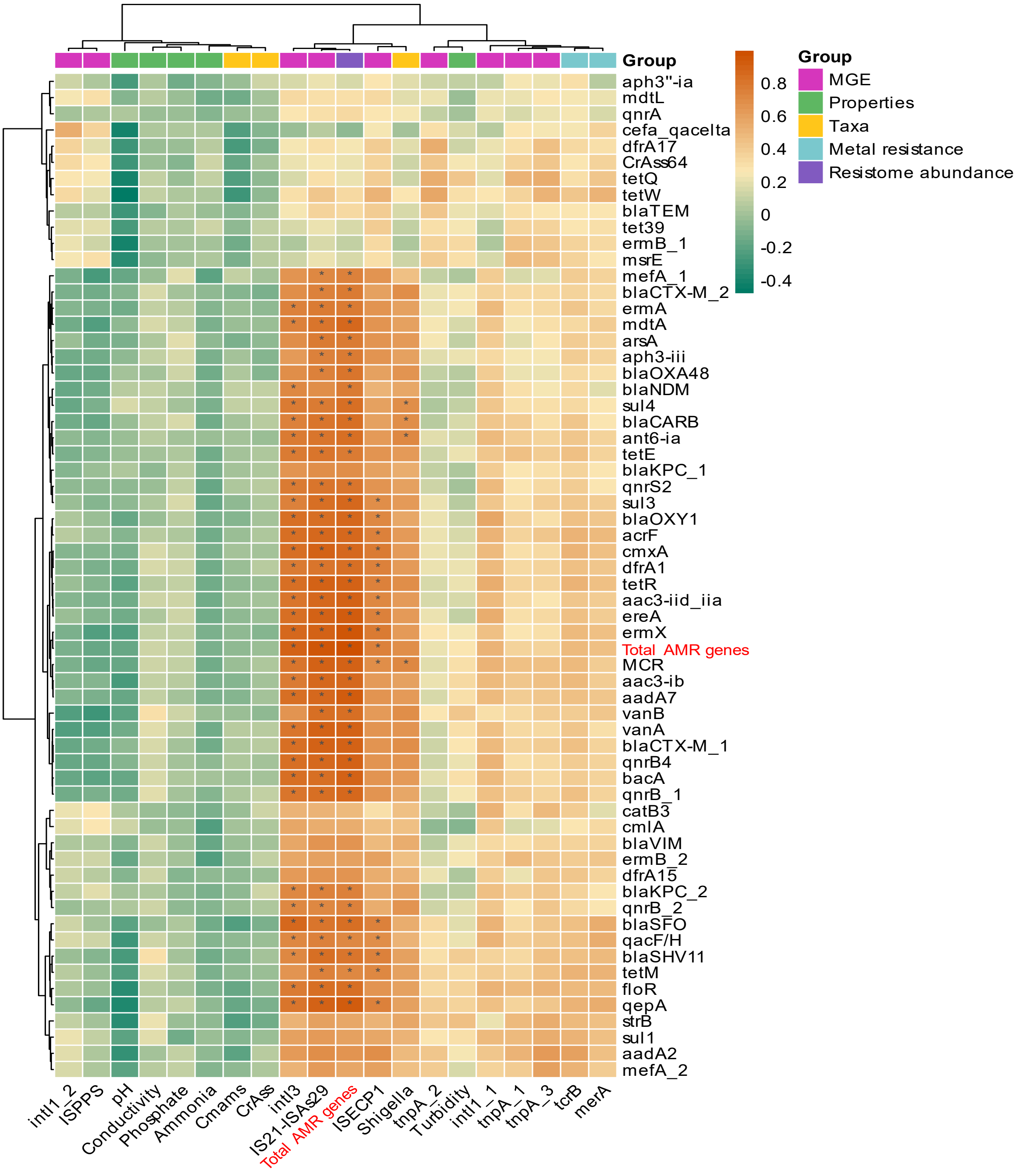

Supplement: Supplementary file 1 [file qvaf013_Supplemental_Files.zip › IAV_AMR_Pond_Supplementary_V4_Figure2.pdf]
